# Identification of Association Between Mucus Microbiota and Gene Expression in the Gill of a *Streptococcus agalactiae*-Resistant Nile Tilapia Strain Though Multi-Omics Analyses

**DOI:** 10.3390/ani16091389

**Published:** 2026-05-02

**Authors:** Haotian Wu, Liting Chen, Ting Huang, Yi Liu, Liming Luo, Lingyu Kong, Yumeng Sun, Wenchao Zhang, Zhongbao Guo, Zhichang He, Yongju Luo, Jun Xiao, Ying Lu

**Affiliations:** 1 Key Laboratory of Exploration and Utilization of Aquatic Genetic Resources, Ministry of Education, Shanghai Ocean University, Shanghai 201306, China; wht10001@126.com (H.W.); yiliu1013@126.com (Y.L.); luoliming0104@live.com (L.L.); sallykong001230@outlook.com (L.K.); 18121070935@163.com (Y.S.); 2 International Research Center for Marine Biosciences, Ministry of Science and Technology, Shanghai Ocean University, Shanghai 201306, China; 3 Guangxi Key Laboratory of Aquatic Genetic Breeding and Healthy Aquaculture, Guangxi Academy of Fishery Science, Nanning 530021, China; chenlt3433@126.com (L.C.); htwish@163.com (T.H.); 18833516221@163.com (W.Z.); guozhongbaono1@163.com (Z.G.); he_zhichang@outlook.com (Z.H.); lfylzc123@163.com (Y.L.); 4 College of Life Sciences, Hunan Normal University, Changsha 410081, China

**Keywords:** tilapia, *Streptococcus agalactiae*, 16S rRNA sequencing, transcriptome, metabolome, microbiota

## Abstract

*Streptococcus agalactiae* infections severely threaten global tilapia aquaculture, causing substantial mortality and economic damage. The “Zhuangluo 1” (ZL) strain, derived from the fast-growing GIFT Nile tilapia and refined through multiple generations of selection, uniquely combines robust resistance to *S. agalactiae* with improved growth traits. This study examined gene expression and regulation of gill mucus microbiota in ZL during experimental *S. agalactiae* challenge. 16S rRNA sequencing revealed *Flavobacterium*, *Vogesella*, *Hydrogenophaga*, *Acidovorax*, *Rheinheimera*, and *Deinococcus* as prominent genera in the gill mucus microbiome of ZL across time points. Transcriptome time-course analysis identified differentially expressed genes in gills of ZL that were predominantly enriched in cytoskeleton in muscle cells and motor protein pathways. Abundances of the dominant genera *Flavobacterium* and *Hydrogenophaga* showed significant correlations with genes regulating mucus secretion, mucin glycosylation, immune modulation, and oxidative stress response in ZL. Untargeted metabolomics of gill mucus revealed substantially higher levels of metabolites potentially linked to microbial metabolism and host–microbiota interactions in ZL. A complementary genome-wide association study for resistance in ZL further localized genes underlying these expression–microbiota associations. These findings elucidated microbiota–host interactions between ZL and gill mucus microbiota, and provide more insights into the role of mucus regulation in disease resistance.

## Simple Summary

Tilapia is a major global aquaculture species, but *Streptococcus agalactiae* infections severely limit its farming. The “Zhuangluo 1” (ZL) strain is the first inbred tilapia line combing strong resistance to *S. agalactiae* and excellent growth performance, which is derived from the fast-growing GIFT Nile tilapia and refined through over five generations of pedigree-based selection for disease resistance. This study investigated microbiota of mucus on gills during an experimental challenge of *S. agalactiae* in ZL, which revealed that certain bacterial groups, such as *Flavobacterium* and *Hydrogenophaga*, were more abundant in the gill mucus of resistant individuals. These changes were closely linked to expression of the host genes that regulated the mucus composition and immune responses, implicating a potential host–microbe interaction. A genetic study further localized the candidate genes involved in the regulation. Overall, the ZL strain was able to effectively shape its gill mucus microbiota, providing new insights into natural disease resistance. This knowledge will contribute to breeding the healthier tilapia strains, reduce reliance on antibiotics, and support more sustainable aquaculture for global food production.

## 1. Introduction

Tilapia is one of the most farmed fishes and is currently produced in over 150 countries. However, tilapia is highly susceptible to *Streptococcus agalactiae* infection [1]. In extreme cases, the mortality rate of diseased fish exceeds 90*%* [2]. *S. agalactiae* has been reported to infect tilapia primarily via three routes. The first is through waterborne transmission. Healthy fish get infected though gills during respiration or osmoregulation. Secondly, healthy fish ingest bacteria-contaminated feed or diseased fish, and the bacteria infect the fish though intestinal epithelium. Finally, healthy fish get infected though injury and direct contact with diseased fish [3,4]. These three routes reveal that gills and intestinal epithelium play an important role in *S. agalactiae* infection. They both have a microenvironment supporting abundant microbiota, but correlation between these microbiota and *S. agalactiae* remains unknown.

The genetically improved farmed tilapia (GIFT) strain, which originates from Nile tilapia (*Oreochromis niloticus*), is one of the most successful selective breeding programs in aquaculture [5]. It has become the foundation of many subsequent breeding programs worldwide. The GIFT strain is the predominant commercial tilapia line in global aquaculture; however, it is highly susceptible to *S. agalactiae* infection, with reported mortality rates reaching up to 90% [6]. Such high mortality frequently causes substantial economic losses for farmers [7]. Although several breeding programs have been initiated to improve disease resistance in GIFT, few have achieved notable success. Zhuangluo 1 (ZL) is the first disease-resistant strain developed from GIFT through family-based selection. The ZL breeding program began in 2003, with resistance to *S. agalactiae* as the primary target trait [8]. After more than five generations of family selection, the average mortality rate of ZL against *S. agalactiae* dropped to as low as 5%. Moreover, the absolute growth rate of ZL reached 2.50 g per day, which is higher than the 1.89 g per day observed in its founder (F0) GIFT population [9]. Previous studies on ZL failed to explain how ZL acquired resistance to *S. agalactiae* without losing growth traits. Instead, they mainly attributed the disease resistance trait to different gene expression. These differences involve the centrality of the pathogen’s attachment and recognition, cytoskeletal rearrangement, immune activation/inflammation at pathogen entry, and host inflammatory response pathways [10], as well as a stronger ability of pathogen recognition, antigen presentation, and immune activation [11]. Recent research discovered that some microbiota characteristics of ZL are heritable [12]. These findings prompted us to further investigate the relation between *S. agalactiae* infection and ZL’s microbiota.

It is difficult to acquire a breeding strain featuring both growth and disease resistance. In most cases, creatures have to make a trade-off between immune function and growth, owing to physiological limitations or energy resource management [13]. A compromise in immune function was observed after selection for growth in poultry [14]. The immune system component MHC (Major Histocompatibility Complex) is associated with body weight, egg weight, egg laying intensity, and weight of sexual maturity in chickens [15]. However, in certain situations, animals can acquire pathogen resistance through modulation of their external microbiota. This strategy allows animals to acquire immunity to certain pathogens through modulation of their associated microbiota rather than solely through their own immune system [16,17]. Such microbiota-mediated resistance provides a potentially attractive model for simultaneously improving immune function and growth traits in a single breeding strain. It has been reported that a healthy intestinal flora could improve host immunity by preventing colonization or cut down the nutrition absorption of pathogens [18,19,20]. Likewise, hosts may also remodel their microbiota to achieve resistance to environmental toxins [21]. A systematic study on how organisms acquire immunity to specific pathogens by regulating outside microbiota could fill the gap in this field and create new research directions for animal breeding. Unfortunately, few researchers have carried out such study.

In this study, we conducted 16S rRNA sequencing to investigate the gill mucus microbiota of the resistance ZL strain during an *S. agalactiae* challenge. Furthermore, we examined the gill transcriptome and its correlation to abundance of gill mucus microbiota. Adding metabolic findings from gill mucus, we revealed a dynamic network of host gene expression, correlated mucus microbiota and responsible metabolites. A parallel genome-wide association study provides further evidence on the correlated genes and their association with *S. agalactiae* resistance.

## 2. Materials and Methods

### 2.1. Cultivation of Streptococcus agalactiae GD001 Strain

The *S. agalactiae* strain GD001 used in the challenge was presented by the Fish Disease Prevention and Control Laboratory in the Guangxi Academy of Fishery Sciences. The whole 16S rRNA sequence of GD001 has 100% identity to the *S. agalactiae* strain SA20-06 [22]. The strain stored at −80 °C was retrieved and thawed, followed by initial inoculation on Columbia Agar Base Columbia Agar (Cat. No. B682017; Sangon Biotech Co., Ltd., Shanghai, China) for amplification. The inoculated plates were then incubated in a constant-temperature incubator set at 28 °C for 24 h. The resultant colonies on the plates were thoroughly washed and diluted with phosphate-buffered saline (Cat. No. E607008; Sangon Biotech Co., Ltd., Shanghai, China). For strain rejuvenation, individuals of sensitive GIFT were intraperitoneally injected with 0.2 mL of GD001 strain (about 1 × 10^9^ CFU/mL) and each separated into heated buckets at 32 °C. Pathogenic bacteria were subsequently isolated from dying fish brain and inoculated to healthy fish again. This process was repeated until the bacteria caused the sensitive GIFT tilapia mortality rate to reach over 80% after intraperitoneal injection.

### 2.2. Measurement of Semi-Lethal Concentrations for the GD001 Strain

Resistant ZL and susceptible GIFT tilapia were provided by the National Tilapia Seed Farm in Nanning, Guangxi, China. Healthy and uninjured tilapia were carefully selected and then acclimatized and cultured under the experimental condition (pH 7.1–7.5, temperature 32 °C) for two weeks prior to the experiment in tanks separated by webs. They were fed with commercial pellets by 2% of body weight per day twice, which was stopped 48 h prior to the challenge experiment. Individuals for experiments weighted 50 ± 5 g.

To determine the final semi-lethal concentration (LD_50_) for the strain GD001, five experimental groups were set under the concentration gradients of 1.0 × 10^5^, 1.0 × 10^6^, 1.0 × 10^7^, 1.0 × 10^8^, and 1.0 × 10^9^ CFU/mL (estimated by a spectrophotometer). Each group includes 20 GIFT individuals, intraperitoneally injected with 0.2 mL of strain GD001. The treated fish were grown in a 100 L water bucket for routine maintenance by changing 50% of water every other day. The water temperature was meticulously kept at 32 °C. Mortality rates were recorded by enumerating deceased fish every 2 h throughout the experimental duration. The final LD50 for the strain GD001 is determined as 5.83 ×10^8^ CFU/mL by the Reed–Muench method [23], which was used in the following challenge experiment.

### 2.3. Challenge Experiment and Sample Collection

Limited by laboratory facilities and efficiency of plate cultivation, intraperitoneal injection was adopted for the challenge experiment. This approach was also deliberately chosen to achieve uniform infection progression and synchronized sampling time points, which are difficult to obtain via mucosal challenge routes due to high inter-individual variability. The intraperitoneal injection of *S. agalactiae* was carried out on 60 ZL and 60 GIFT tilapias, using 0.2 mL of GD0001 strain under 5.8 ×10^8^ CFU/mL per fish. The first death occurred at 6 h post-infection. The mortality rate exhibited a rapid rise until around 12 h and slowed down afterwards. Post 48 h, only a few deaths were observed every day.

Samples were collected at 4 time points, 0 h, 12 h, 24 h and 48 h post-infection. For gill mucus and intestine (hindgut) content, pooled samples were collected from 10 individuals with swabs. All pooled samples have at least three biological replicates for both groups at the 4 time points. An exception is the gill mucus sample from ZL at 0 h, where we lost one pooled replicate due to a transportation accident. Gill tissues were also collected at 4 time points with three biological replicates.

### 2.4. Sequencing of 16S rRNA V3 and V4 Regions

Pooled samples of gill mucus and intestine content were involved in 16S rRNA sequencing. After DNA extraction, V3-V4 high variant regions of 16S rRNA were amplified by PCR using general primers, 5′-ACTCCTACGGGAGGCAGCAG-3′ and 5′-GGACTACHVGGGTWTCTAAT-3′. Samples with DNA content below 200 ng were excluded from further analysis.

The constructed libraries with an average insertion size of 350 to 400 bp were sequenced on the Illumina MiSeq platform to generate raw reads. Adapt sequences were removed using Cutadapt software (v 4.5). [24]. The paired-end sequences were merged with PEAR (v 5.36.1) [25]. PRINSEQ (0.20.4)[26] was used in the quality control process, while Usearch (v 11.0.667) [27,28] was chosen for operational taxonomic unit (OTU) clustering at the similarity threshold of 97%.

### 2.5. Statistical Analysis for 16S rRNA Sequencing Data

Taxonomies of the microbiota were identified by searching the Ribosomal Database Project (RDP) Classifier (www.glbrc.org/data-and-tools/glbrc-data-sets/ribosomal-database-project (accessed on 15 May 2023)) [29]. The R packages “ade4 (v 1.7-22)”, “cluster (v 2.1.4)”, and “clusterSim (v 0.51-1)” were adopted for classification of microbiota types. The rarefaction analysis and alpha-diversity indices were calculated with Mothur (v 1.48.0) [30]. Prior to parametric testing, the assumptions of normality (Shapiro–Wilk test) and homogeneity of variances (Levene’s test) were examined. Shannon and Pielou’s evenness (Shannoneven) indices met the assumptions of normality (*p* > 0.05) and homogeneity of variances (*p* > 0.05). Simpson’s index violated the normality assumption (*p* < 0.001) but showed homogeneous variances. Therefore, the effects of challenge time (GIFT vs. ZL) and their interaction on Shannon and Shannoneven were analyzed using two-way ANOVA (fixed factors: time and group, including the time × group interaction term). When significant effects or interactions were detected (*α* = 0.05), post hoc pairwise comparisons were performed with Student’s *t*-test (or Welch’s *t*-test if needed) and Benjamini–Hochberg FDR correction for multiple comparisons. For Simpson’s index, because of non-normality, a nonparametric aligned rank transform (ART) two-way ANOVA was used as the primary analysis.

Microbial beta-diversity was assessed using non-metric multidimensional scaling (NMDS) ordination of Bray–Curtis dissimilarities calculated at the genus level (stress = 0.0964). Differences in community composition among groups were tested with PERMANOVA using all possible permutations (v egan v2.6-4 R package). In addition, sparse partial least-squares discriminant analysis (sPLS-DA) was performed using the mixOmics(v 6.22.0) [31] R package to explore potential discriminatory features. Given the small sample size in one group (*n* = 2), the optimal number of components was set to 2, and the number of variables retained per component was determined via tune.splsda function. Model performance was evaluated using leave-one-out cross-validation, and overall significance was assessed with a 500-permutation test of group labels.

Linear discriminant analysis effect size (LEfSe) was performed for biomarker discovery using the microeco(v **0.17.0**) R package [32]. Kruskal–Wallis rank-sum tests were applied to identify taxa with differential abundance across groups (*α* = 0.05), without further *p*-value adjustment. Taxa passing the adjusted *p*-value threshold then underwent linear discriminant analysis (LDA) to estimate effect sizes. An LDA score threshold of ≥3.0 was applied (more stringent than the common default of ≥2.0) to select discriminative features. LEfSe results were visualized with cladograms and bar plots, selecting the 100 most significant features and the 200 most abundant taxa as background. Other basic analysis and plotting are performed with R language.

### 2.6. Gill Transcriptome Sequencing and Data Analysis

Total RNA of gill tissue was extracted using a TRIzol reagent (Cat. No. B610409; Sangon Biotech Co., Ltd., Shanghai, China). RNA sequencing was performed on the Illumina HiSeq × Ten platform. Cutadapt(v 4.5) [24] was used to remove sequencing connectors and low-quality reads. Clean reads were mapped to the GIFT strain tilapia genome (Genbank Accession No. GCA_922820385.1) ([33] by STAR (v 2.7.10b) [34]). The gene expression was calculated by featureCounts(v 2.0.6) [35], normalized to FPKM with R script. A screening workflow was applied to identify the differentially expressed genes (DEGs) in ZL. Briefly, among the ZL genes with significantly higher (or lower) expression at 12, 24, or 48 h post-infection than uninfected ZL (0 h), those also having a significantly higher (or lower) expression than the GIFT control at the corresponding time point were defined as the ZL DEGs (Figure S1). The significantly differential expression of all the genes was determined by the R package “DEGseq (v 1.52.0)” [36] with the following parameters: adjusted *p*-value (FDR) < 0.05 and |log_2_ fold-change| > 2. The R package “Mfuzz”(v 2.60.0) [37] was used for time-course cluster analysis of gene expression profiles. The fuzzifier parameter (m) was estimated as 1.50482 using the mestimate() function. The optimal number of clusters was determined to be 8 using the Dmin() function by identifying the elbow point in the minimum centroid distance plot. Gene functions were annotated by an enrichment analysis with Kyoto Encyclopedia of Genes and Genomes pathway database (KEGG) [38] via R package “clusterProfiler”(v 4.6.2) [39]. The enrichment analysis was performed with the following parameters: qvalueCutoff = 0.2, pvalueCutoff = 0.05, and pAdjustMethod = “BH”.

### 2.7. LC-MS/MS Metabolomics Analysis

The LC/MS system consisted of a Waters Acquity I-Class PLUS ultra-high-performance liquid chromatograph (UHPLC) coupled to a Waters Xevo G2-XS QTOF high-resolution mass spectrometer (Waters Corporation, Milford, MA, USA). Separation was performed on a Waters Acquity UPLC HSS T3 column (1.8 μm, 2.1 × 100 mm). Collected gill mucus samples (see Section 2.3) were injected with a 2 μL aliquot. The mobile phase consisted of (A) 0.1% formic acid in water and (B) 0.1% formic acid in acetonitrile for both positive and negative ion modes. Mass spectrometry data were acquired in MSE mode using MassLynx V4.2 software (Waters). In each acquisition cycle, low-collision-energy (4 eV or off) and high-collision-energy (ramped from 10 to 40 eV) spectra were collected alternately, with a scan time of 0.2 s per spectrum. The ESI ion source was operated with a capillary voltage of +2.5 kV in positive ion mode and −2.0 kV in negative ion mode, a cone voltage of 30 V, and temperatures of 100°C (source) and 500°C (desolvation). Gas flows were set to 50 L/h (cone) and 800 L/h (desolvation).

Raw data were processed with Progenesis QI (v 3.0) software for peak detection, alignment, and normalization. Compounds were identified by searching against the METLIN database (v ia Progenesis QI) [40] and an in-house Biomark library. Peak areas were normalized to the total peak area prior to downstream analysis. Intra-group repeatability and quality control (QC) sample stability were evaluated using principal component analysis (PCA) and Spearman correlation analysis. Identified metabolites were annotated for classification and pathway information using the KEGG, HMDB, and LIPID MAPS databases. Fold changes between groups were calculated, and statistical significance was assessed using Student’s *t*-test (*p*-value).

### 2.8. Gene–Microbial Correlation Analysis

The gene–microbial correlation analysis involved 663 ZL DEGs described in 2.6, and the 27 most abundant bacteria genera (maximum abundance exceed 0.5%). As gill mucus samples were pooled across individuals at each sampling time point, the mean relative abundance of each bacterial genus and the mean expression level of each gene were calculated per time point and used for all subsequent correlation analyses. Pearson correlation and Student’s *t*-test were adopted to evaluate the relevance of gene expression and bacteria abundance. In order to acquire ZL-specific positive correlated gene–bacteria pairs, GIFT positively correlated pairs (coefficient > 0.7) were excluded ahead of time. Later on, we selected significantly positive ZL pairs by coefficient > 0.7 and *t*-test *p* < 0.05. These ZL-specific positive correlations were optimized and visualized by Cytoscape (v 3.9.1, [41]), in addition to acquiring gene–gene interaction knowledge from STRING database (v 12.0, [42,43]). The statistics were calculated by R software (v 4.2.3) while the original network was generated by Igraph package (v 1.5.1, [44]).

### 2.9. Genome-Wide Association Analysis on Streptococcus agalactiae Resistance

Tail fin samples were collected from 141 ZL tilapia that survived a natural *S. agalactiae* outbreak and 150 ordinary GIFT tilapia as controls. Genomic DNA was extracted and subjected to whole-genome resequencing at an average depth of 5.9 ×. Reads were aligned to the tilapia reference genome using BWA-MEM [45], and variants were called with GATK [46]. After quality filtering (minor allele frequency > 0.05, missing rate < 0.1, and other standard criteria), genotypes were imputed with Beagle [47]. Genome-wide association analysis for survival status (binary trait) was performed using Efficient Mixed-Model Association eXpedited (EMMAX)(beta version, 07Mar2010) [48] to account for population structure and relatedness. Manhattan and quantile–quantile plots were generated with the CMplot(v 4.5.1) [49] R package to visualize significant associations. Full details of the bioinformatics pipeline, filtering criteria, and software versions are provided in the Supplementary Methods.

### 2.10. Ethics Statement

All procedures involving animals in this study were approved by the ethics committee of laboratory animals of Shanghai Ocean University under the protocol number SHOU-DW-2023-094. Gill mucus samples were collected non-invasively via gentle swabbing. Other sampled individuals were euthanized by anesthetic overdose of MS-222 (tricaine methanesulfonate) at 50 mg/L for a minimum of 25 min. Death was confirmed by cessation of opercular movement, absence of tail pinch reflex, and loss of vestibulo-ocular reflex. The euthanasia approach is consistent with the Laboratory animal—Guidelines for euthanasia (GB/T 39760-2021) [50].

## 3. Results

### 3.1. Tilapia Strains and S. agalactiae Challenge Trial

**Figure 1:**
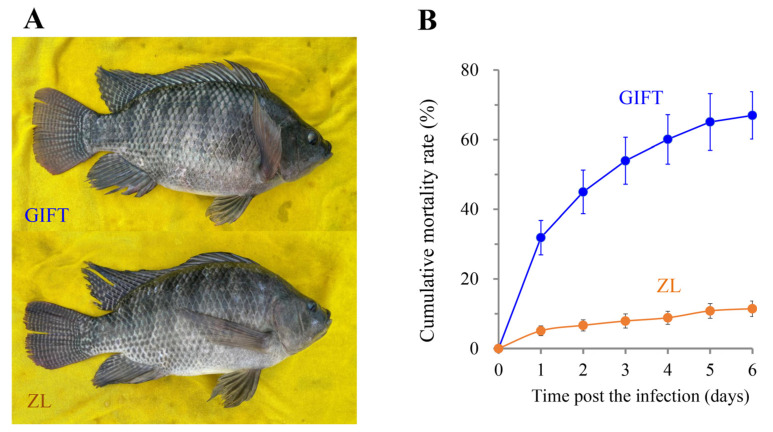
Sequenced tilapia strains and mortality rate after infection. (**A**) Images of GIFT and Zhuanluo 1 (ZL) adult males. (**B**) Observed mortality rate during the period of *S. agalactiae* challenge.

ZL is an inbred tilapia strain derived from GIFT (Figure 1A) that exhibits outstanding resistance to *S*. *agalactiae*. In an independent challenge trial, final mortality reached 66.98% in GIFT, whereas only approximately 10% of ZL individuals succumbed under identical conditions (Figure 1B).

### 3.2. 16S rRNA Sequencing of Pooled Gill Mucus and Intestine Content

**Figure 2:**
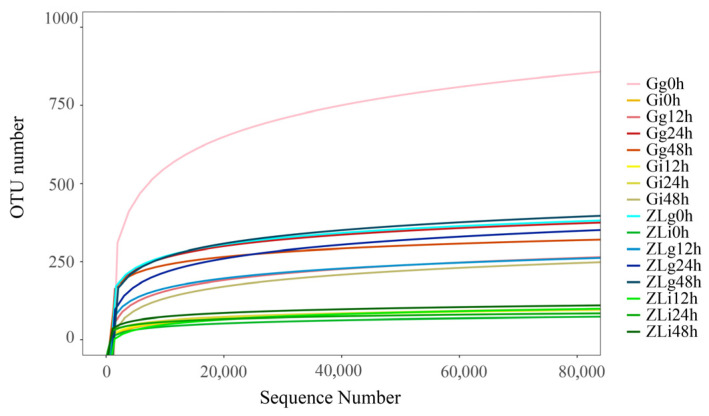
Rarefaction curves illustrating the relationship between the number of clustered operational taxonomic units (OTUs) and sequencing read depth in different sample groups. GIFT tissues are represented by red and yellow lines, while ZL tissues are indicated by blue and green lines. Sample abbreviations: G, GIFT; g, gill mucus; i, intestine contents.

Sequencing of the V3-V4 region of the 16S rRNA gene generated a total of 5,457,869 reads, with an average of 109,157 reads per sample. The average read length was 375 bp, and all libraries achieved >99.9% coverage. These sequences were clustered into 2179 operational taxonomic units (OTUs) at 97% similarity. The gill mucus samples contained substantially higher bacterial diversity than the intestine content samples. Specifically, the number of OTUs in gill mucus was approximately 3.8 times higher than in the intestine contents. OTU numbers in the intestine content samples remained almost constant throughout the *S. agalactiae* challenge period. Rarefaction curves for each sample approached a plateau (Figure 2), indicating that the sequencing depth was sufficient to capture most of the bacterial community diversity.

### 3.3. Variation in Microbiota Diversity in Gill Mucus During S. agalactiae Challenge

Sequencing of the 16S rRNA amplicons revealed a significantly higher number of OTUs in gill mucus than in intestine contents. In addition, the OTUs in gill mucus showed more pronounced variations during challenge (Figure 2). Since intraperitoneal injection provides less influence on intestinal microbiota (the intestinal analysis results are less varied and of less relevance to this manuscript), they are provided in the Supplementary Materials.

**Figure 3:**
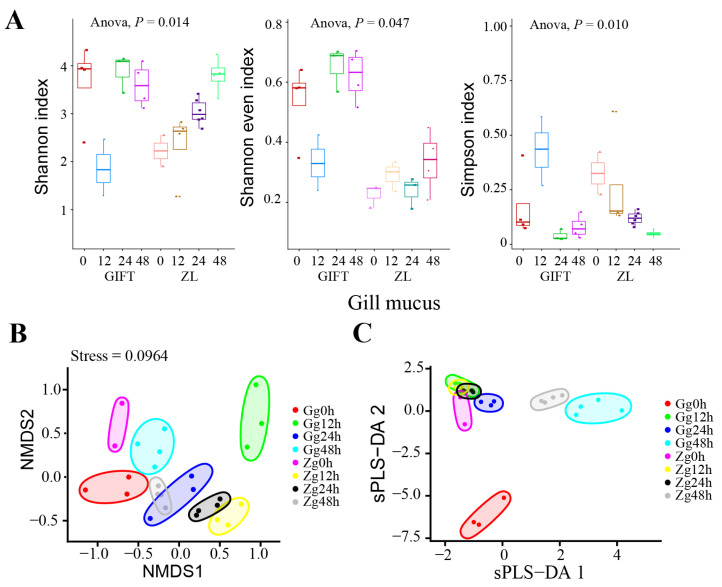
Microbial alpha- and beta-diversity in gill mucus during *S. agalactiae* challenge. (**A**) Box plots of microbial alpha-diversity indices (Shannon index, Shannon evenness index, Simpson index) in the gill mucus of the two tilapia strains at 0, 12, 24, and 48 h post-challenge. The *P*-values from ANOVA reflect significant differences in alpha-diversity among groups. (**B**) Non-metric multidimensional scaling (NMDS) ordination, illustrating beta-diversity and microbial community structure in gill mucus. (**C**) Partial least-squares discriminant analysis (PLS-DA) score plot, showing differentiation in microbial community structure between tilapia lines and across time points in gill mucus. The sample identifications are abbreviated thus in (**B**,**C**): G for GIFT; Z for ZL; g for gill mucus.

Alpha-diversity in gill mucus showed a significant group × time interaction for the Shannon–Wiener index (two-way ANOVA: *F* (3,22) = 4.43, *P* = 0.014) and Shannon evenness (*F* (3,22) = 3.12, *P* = 0.047) analyses. A significant group × time interaction (*F* (3,22) = 4.79, *P* = 0.010) was also revealed in the Simpson’s index analysis of log-transformed data. In GIFT fish, Shannon diversity dropped sharply at 12 h post-infection (mean = 1.87) from the 0 h baseline (mean = 3.65), followed by a rapid recovery at 24 h (mean = 3.89) and 48 h (mean = 3.60) (Figure 3A). In contrast, ZL fish displayed a gradual, non-significant increase in Shannon diversity from 2.23 at 0 h to 3.81 at 48 h. The Shannon evenness and Simpson’s index both followed a closely parallel temporal pattern. Post hoc comparisons with the Benjamini–Hochberg FDR correction confirmed the significant decline in GIFT fish at 12 h (*P*_shannon_ = 0.022). By 48 h, all three indices (Shannon, Shannon evenness, and Simpson) converged to similar levels in both the GIFT and ZL groups.

These findings indicate that an increase in gill mucus microbiota diversity after the initial infection phase is a common feature among surviving tilapia. The rapid twofold recovery of alpha-diversity in GIFT fish from 12 h to 48 h coincided with slowing mortality, while a similar proportional increase occurred more steadily in the resistant ZL group throughout the period. This shared dynamic may be associated with resistance to *S. agalactiae* infection.

Microbial beta-diversity was assessed by NMDS ordination of Bray-Curtis dissimilarities at the genus level (stress = 0.0964, Figure 3B). PERMANOVA revealed overall significant differences in genus-level community composition among groups (pseudo-*F* = 11.56, *R*^2^ = 0.82, *P* = 0.0001). However, after Benjamini–Hochberg correction for multiple testing, no individual pairwise comparisons remained statistically significant, likely due to the large number of tests performed and consequent reduction in statistical power. Nevertheless, the NMDS ordination showed a subtle temporal trend within the ZL and GIFT series, with limited separation between the two series except in Gg12h.

An sPLS-DA model (ncomp = 2) was additionally applied as an exploratory supervised approach (Figure 3C). The score plot displayed visible group clustering. Leave-one-out cross-validation, however, yielded a high balanced error rate (BER = 0.75 using max.dist). A permutation test (500 permutations) indicated that the observed classification performance was significantly better than chance (*p* < 0.002). Due to the very small sample size in one group (*n* = 2) and the relatively high cross-validated error rate, this supervised analysis carries a substantial risk of overfitting despite variable selection. Therefore, interpretation of beta-diversity patterns primarily relies on the unsupervised NMDS ordination and the PERMANOVA test, while the sPLS-DA results are presented mainly to highlight candidate discriminatory genera.

**Figure 4:**
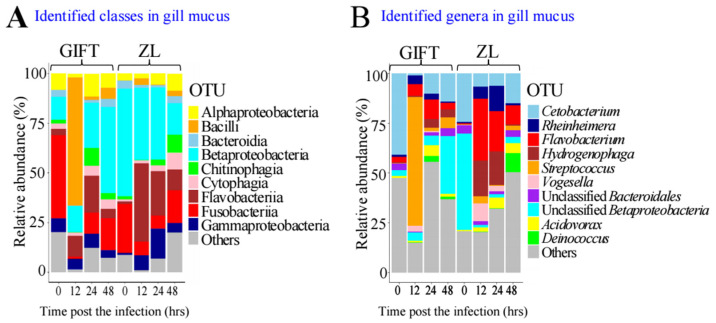
Composition and relative abundance of microbiota in gill mucus and intestine contents. (**A**,**B**) Identified classes and genera in gill mucus, respectively. Microbiota with less than 5% abundance were categorized as “others”.

To further explore the drivers behind these diversity shifts, we next examined the taxonomic composition of the gill mucus microbiota at the class and genus levels. At 0 h, GIFT contained 13 bacterial classes with relative abundance > 1%, which decreased to seven at 12 h, and recovered to 13 classes by 24–48 h. In contrast, ZL contained 10 bacterial classes with relative abundance > 1% at 0 h, eight classes at 12 h, nine classes at 24 h, but 14 classes at 48 h. Furthermore, the gill mucus of the GIFT control group, pre-challenge, contained Fusobacteriia (41.90*%*), Betaproteobacteria (11.53*%*), and Alphaproteobacteria (8.30*%*). However, the gill mucus of ZL pre-challenge (Zg0h) predominantly contained Betaproteobacteria (54.10*%*), Fusobacteriia (24.65*%*), and Bacteroidia (4.15*%*) (Figure 4A; Table S1). The abundance of Fusobacteriia in GIFT was nearly four times that of Betaproteobacteria, whereas in ZL, Betaproteobacteria exceeded Fusobacteriia by more than twofold. At 12 h in GIFT, the class Bacilli (which includes *S. agalactiae*) surged to 64.37%, dominating the community and causing distinct clustering in the beta-diversity analysis. Subsequently, Betaproteobacteria abundance in GIFT rose progressively (13.13% at 12 h → 22.77% at 24 h → 43.50% at 48 h), coinciding with Bacilli dropping to 1.53% by 24 h. However, ZL showed elevated Flavobacteriia abundance (39.40% at 12 h and 22.30% at 24 h) instead of the Bacilli observed in GIFT. This suggests that the presence of elevated Betaproteobacteria and Flavobacteriia may relate to a healthy robust gill mucus microbiota.

Genus-level analysis confirmed massive *S. agalactiae* accumulation in GIFT early in infection (peaking at 64.36% at 12 h), followed by a sharp decline at 24–48 h (Figure 4B; Table S2), consistent with the observed diversity shifts. In ZL, *S. agalactiae* remained <4% throughout, resembling the recovery phase in GIFT. Again, very low *S. agalactiae* levels (in GIFT at 48 h and ZL at 0 h) coincided with high Betaproteobacteria levels (43.50% and 54.10%).

Although unclassified Betaproteobacteria decreased during infection, certain Betaproteobacteria genera increased, including *Hydrogenophaga*, *Vogesella*, and *Acidovorax*. Even at 12 h, Betaproteobacteria in ZL retained 36.30% abundance and remained stable at 24–48 h. Changes in other classes were often driven by single dominant genera: increased *Fusobacteriia*, *Flavobacteriia*, and Gammaproteobacteria linked to *Cetobacterium*, *Flavobacterium*, and *Rheinheimera*. Among these, *Flavobacterium*, *Rheinheimera*, and *Hydrogenophaga* showed marked differences between strains. In ZL gill mucus at 12h, these aerobic genera totaled 54.96% abundance, compared with only 11.02% (12 h) and 17.04% (24 h) in GIFT.

### 3.4. Identification of Featured Biomarkers in ZL Tilapia

**Figure 5:**
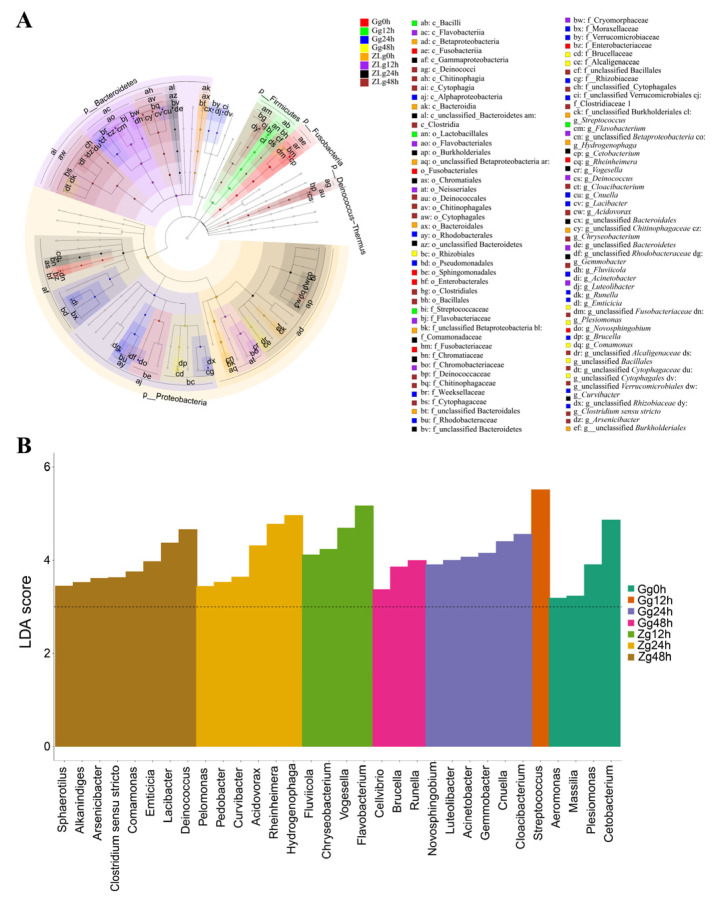
Linear discriminant analysis effect size (LEfSe) of microbial community differences between groups. (**A**) Cladogram showing the hierarchical structure of microbial taxa from phylum to genus. Concentric circles (from inner to outer) represent taxonomic ranks, with the diameter of each circle proportional to the relative abundance of the taxon. Clade color indicates the group with which the taxon is associated: taxa enriched in different groups are distinguished using highly contrasting colors (e.g., red for Ggoh). To avoid overlapping issues caused by the hierarchical branching structure of the cladogram, clades are drawn in lighter shades of the corresponding group colors. The color of each node and its accompanying letter label together identify the characteristic bacterial taxa.. The sample identifications are abbreviated thus: G = GIFT strain, Z = ZL strain, g = gill mucus. (**B**) Histogram of linear discriminant analysis (LDA) scores (log_10_-transformed) for differentially abundant taxa. Only taxa with an LDA score ≥ 3.0 are shown. The length of each bar represents the magnitude of the effect size, with longer bars indicating stronger differential enrichment between groups.

Linear discriminant analysis effect size (LEfSe) was used as an exploratory tool to identify candidate bacterial genera discriminating between ZL and GIFT tilapia during the *S. agalactiae* challenge. This analysis identified 32 genera with LDA scores ≥ 3.0, including 9 genera that exceeded 5% relative abundance during the challenge (Figure 5A,B). These genera played a prominent role in the gill mucus microbiota owing to their high abundance. They comprised *Cetobacterium* (featured in Gg0h), *Streptococcus* (Gg12h), *Cloacibacterium* (Gg24h), *Flavobacterium* (Zg12h), *Vogesella* (Zg12h), *Hydrogenophaga* (Zg24h), *Rheinheimera* (Zg24h), *Acidovorax* (Zg24h), and *Deinococcus* (Zg48h). Notably, the following genera were not only featured in ZL but also exhibited substantially higher relative abundances than in GIFT: *Flavobacterium* (12 h: 31.24% vs. 5.99%), *Vogesella* (12 h: 9.34% vs. 3.13%), *Hydrogenophaga* (12 h: 18.02% vs. 0.72%), *Acidovorax* (12 h: 2.14% vs. 0.83%), *Rheinheimera* (24 h: 12.27% vs. 4.45%), and *Deinococcus* (48 h: 9.34% vs. 1.12%). Thus, *Flavobacterium*, *Vogesella*, *Hydrogenophaga*, *Acidovorax*, *Rheinheimera*, and *Deinococcus* emerged as the primary ZL-featured biomarkers with both discriminatory power and elevated abundance during the challenge.

### 3.5. Potential Relationship Between the Changes in Metabolites and Microbiota in Gill Mucus

**Figure 6:**
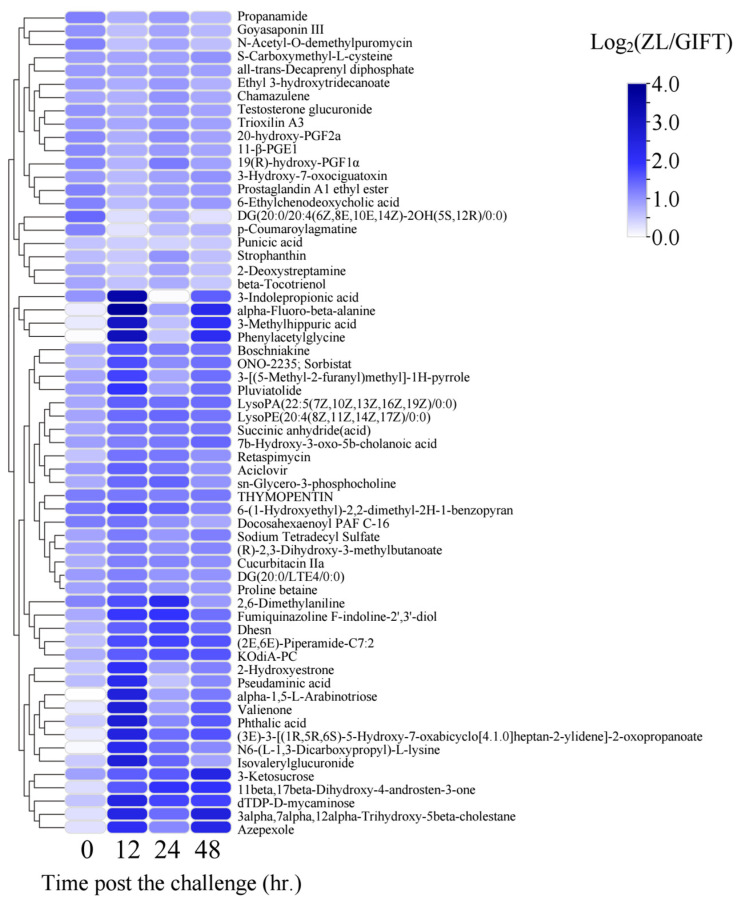
Overview of significantly discriminated metabolites in ZL. Each row and column corresponds to a time point and a metabolite, respectively. The colors indicate the Log_2_ (ZL/GIFT) of the normalized peak area at each corresponding time point (e.g., ZL0h vs GIFT0h, ZL12h vs GIFT12h). The names of the metabolites are shown to the right, and the clustered tree is shown on the left.

Untargeted metabolomics analysis was performed on ZL and GIFT tilapia gill mucus samples to compare metabolite profiles, with particular focus on changes from 0 to 12 h. A total of 62 metabolites showed differential abundance between the two strains at 12 h (Figure 6). Among these, several compounds potentially involved in bacterial carbon and nitrogen metabolism, as well as carbohydrate utilization, showed substantially higher levels in ZL tilapia gill mucus compared with GIFT tilapia (Figure 6). These metabolites included specific organic acids and carbohydrates in the gill mucus of ZL tilapia relative to GIFT at the 12 h time point. They are 3-indolepropionic acid (73.8 vs. 764.0), N6-(L-1,3-dicarboxypropyl)-L-lysine (1469.4 vs. 5422.1), phenylacetylglycine (1188.8 vs. 9550.0), 3-methylhippuric acid (408.5 vs. 2901.4), pseudaminic acid (2013.8 vs. 7583.5), and α-1,5-L-arabinotriose (553.8 vs. 2715.7) (mean peak intensities; GIFT vs. ZL respectively).

### 3.6. Transcriptome Analysis of Gill Samples and Their Association with Gill Mucus Microbiota

**Figure 7:**
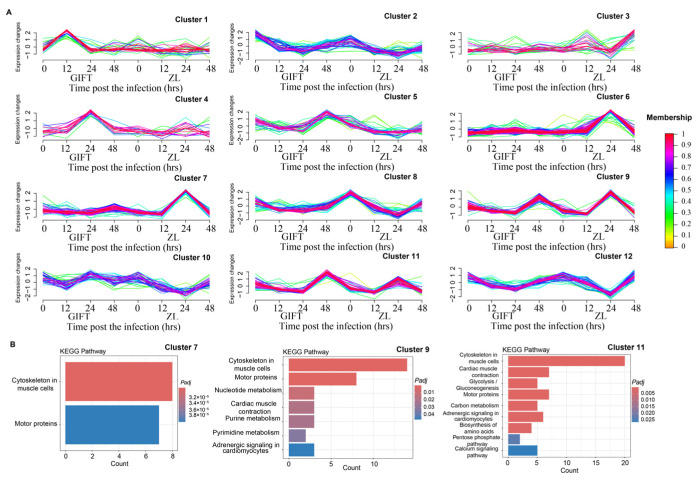
Temporal gene expression profiling and functional enrichment analysis of genes in response to *Streptococcus agalactiae* infection. (**A**) Temporal expression patterns of genes clustered using the Mfuzz algorithm. Genes were grouped into 12 clusters based on the minimum centroid distance (log_2_fold-change) at different time points (0 to 48 h post-infection) in two groups (GIFT and ZL). The color gradient (membership) indicates the membership degree of each gene to its assigned cluster (range: 0 to 1; higher value = stronger membership). (**B**) KEGG pathway enrichment of the DEGs containing at least 5 genes in significantly enriched pathways. Each bar represents a KEGG pathway. The *x*-axis denotes the gene counts, and the *y*-axis lists pathway names, where color intensity correlates with significance level; redder points are more significant.

Following a two-step strategy (Figure S1), we identified 582 ZL-specific DEGs during the *S. agalactiae* challenge (Figure S2). Time-course clustering was performed using the Mfuzz package to discover distinct temporal expression patterns between GIFT and ZL among the 582 DEGs identified (Figure 7A). The optimal number of clusters was determined based on a minimum centroid distance plot, where the rate of decrease in separation stabilized after *K* = 12 (Figure S4). The 582 DEGs were subsequently grouped into 12 distinct clusters, with detailed gene lists for each cluster provided in Table S3.

The clusters exhibited different dynamic profiles. Significantly enriched pathways (adjusted *p*-value < 0.05) containing at least five genes from the cluster were discovered and are briefly discussed here (Figure 7B).

Cluster 7 exhibited a steady expression, except for a high peak in ZL at 24 h. KEGG enrichment highlighted cytoskeleton in muscle cells (*n* = 8 genes) and motor protein (*n* = 7 genes) pathways, pointing to enhanced mucin and other protein traffic within the cells in ZL.

Cluster 9 exhibited a sharp peak in GIFT at 48 h, with an earlier sharp peak in ZL at 24 h. Expression remained low at all earlier time points in both groups. KEGG pathway analysis revealed significant enrichment in cytoskeleton in muscle cell signaling pathway (*n* = 14 genes) and motor proteins (*n* = 8 genes), suggesting involvement in mucin secretion with accelerated kinetics in ZL and a late-phase activation in GIFT.

Cluster 11 was similar to 9, also exhibiting a sharp peak in GIFT at 48 h, with an earlier but lower-amplitude rise in ZL at 24 h. Expression remained low at all earlier time points in both groups. KEGG pathway analysis revealed significant enrichment in cytoskeleton in muscle cells (*n* = 20 genes), cardiac muscle contraction (*n* = 7 genes), motor proteins (*n* = 7 genes), adrenergic signaling in cardiomyocytes (*n* = 6 genes), glycolysis/gluconeogenesis (*n* = 5 genes), carbon metabolism (*n* = 5 genes), and the calcium signaling (*n* = 5 genes) pathways. Genes in this cluster suggested an accelerated coordinated epithelial secretory program in ZL, with a stronger late-phase activation in GIFT.

Clusters 7, 9, and 11 stood out for their ZL-dominant or ZL-accelerated expression dynamics. These clusters, together with their associated pathway enrichments, suggest accelerated or alternative temporal regulatory mechanisms in ZL compared with GIFT, which may partly underly the resistance differences observed between the two strains. In contrast, Clusters 1, 4, and 5 were more closely aligned with GIFT kinetics, but KEGG enrichment did not identify any pathways with ≥5 genes within them.

Building on these strain-specific expression dynamics identified through Mfuzz clustering, we next investigated potential links between host gene expression and temporal shifts in gill mucus microbiota composition in the ZL strain.

In ZL, *Flavobacterium* showed the most pronounced change in relative abundance around the GIFT mortality peak (at 12 h). We identified 118 genes positively correlated with *Flavobacterium* abundance (*R* > 0.7, *p* < 0.05) in ZL, with no equivalent correlations in GIFT (Table S4). These included genes such as *SRP19*, *PPIB*, *FOXL1*, *FABP4A*, *NDUFA1*, and *LTV1* (Table S5).

Also in ZL, *Hydrogenophaga* exhibited the second most significant change in abundance around the GIFT mortality peak (at 12 h), with its relative abundance dynamics showing similar patterns to those of *Flavobacterium*. Similarly, 135 genes were positively correlated with *Hydrogenophaga* in ZL (*R* > 0.7, *p* < 0.05), with no equivalent correlations in GIFT (Table S6). Examples included *HER6*, *CHST3a*, *IL10*, *GPD1c*, and *AK2* (Table S7).

The full lists of correlated genes, together with their functional categorizations (mucus secretion and glycosylation, immune/inflammatory regulation, cellular energy metabolism and mitochondrial function, ribosome biogenesis and mucus content synthesis), are provided in Tables S3–S6.

### 3.7. Host Gene Expression Drives Metabolite Production and Microbiota Remodelling in Gill Mucus

**Figure 8:**
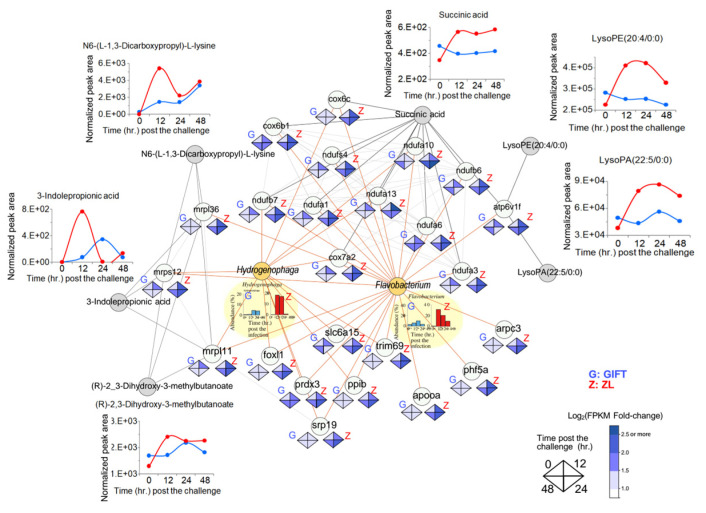
Regulatory network of genes, bacteria, and metabolites in ZL gill samples after *S. agalactiae* challenge. Circles filled in grey, light green, and orange represent the involved genes, metabolites, and microbiota, respectively. Red, light grey, and black lines respectively denote gene–bacteria associations, gene–gene interactions, and gene–metabolite regulations. Expressions of each gene at 0, 12, 24, and 48 h after the challenge are shown in four-color diamonds (clockwise from the top). Relative abundances of *Hydrogenophaga* and *Flavobacterium* are shown below the corresponding circles. The temporal dynamics of the key metabolites are shown beside the corresponding metabolite circles. The network is structured into three functional clusters, a mitochondrial-dominated core on the left, an electron transport chain and energy metabolism cluster on the top right, and a mucus-environment regulatory cluster on the bottom.

Host gene expression, metabolites, and microbiota in ZL gill mucus following *S. agalactiae* challenge were integrated into a multi-layered regulatory network (Figure 8). Genes were grouped into three main functional clusters, with each gene showing correlation(s) to *Flavobacterium*, *Hydrogenophaga*, or both genera (red lines in the Figure 8 plots). Related metabolite changes are displayed adjacent to their corresponding gene clusters.

The cluster on the left was dominated by mitochondrial ribosomal protein genes, including *MRPL36*, *MRPS12*, and *MRPL11*, all significantly upregulated at 12–48 h post-challenge. Metabolite levels of saccharopine (from lysine degradation) and (R)-2,3-dihydroxy-3-methylbutanoate (from valine degradation) increased near this cluster; 3-indolepropionic acid (tryptophan-derived) also rose.

The cluster on the top right contained electron transport chain and energy metabolism genes, including Complex I subunits (*NDUFA3/6/10/13*, *NDUFB6/7*, *NDUFS4*), Complex IV subunits (*COX6B1*, *COX6C*, *COX7B2*), and *ATP6V1F* (v acuolar H^+^-ATPase subunit), all of which were upregulated post-challenge. Succinate levels and the lysophospholipids LysoPE (20:4/0:0) and LysoPA (22:5/0:0) were elevated adjacent to this cluster.

The bottom cluster included mucus-environment regulatory genes FOXL1 (goblet cell differentiation and mucin expression), SRP19 (mucin post-translational modification), and ARPC3 (v esicle transport and exocytosis), which were upregulated during the challenge.

Relative abundances of *Flavobacterium* and *Hydrogenophaga* shifted over 0–48 h (shown below the bacterial nodes). Gene–gene interactions (light grey lines) and gene–metabolite regulations (black lines) connected elements within and across clusters. The network therefore visualized coordinated upregulation of host genes correlated with the two bacterial genera and the associated temporal metabolite dynamics.

### 3.8. Localization of the Resistant Trait in ZL Through a Population-Based GWAS Analysis

**Figure 9:**
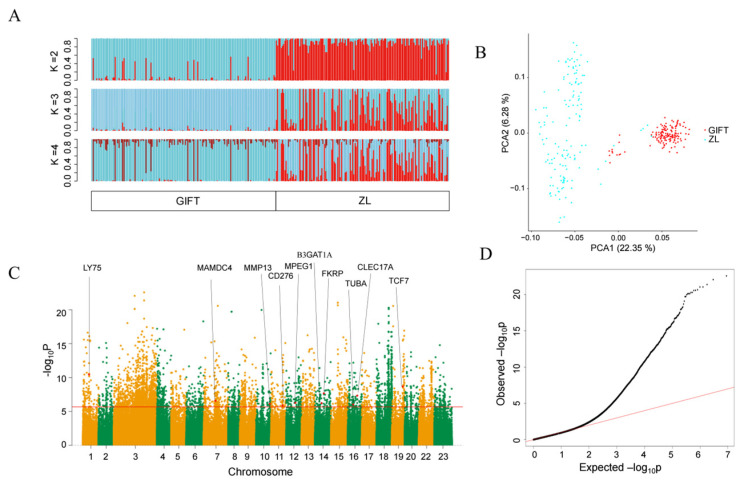
Genome-wide association study (GWAS) for *Streptococcus agalactiae* resistance in tilapia. (**A**) Population structure inferred by ADMIXTURE analysis for ancestral components (*K* = 2–4). Each vertical bar represents one individual, with segment colors indicating the proportion of each ancestral component. Individuals are labeled by population of origin (GIFT or ZL). (**B**) Population structure revealed by principal component analysis. Red dots represent the GIFT strain, and light blue dots represent the ZL strain. PC1 and PC2 explain 22.35% and 6.28% of the variance, respectively. (**C**) Manhattan plot of the GWAS outputs showing genome-wide significance. The *x*-axis shows chromosomal positions across all chromosomes; the *y*-axis shows −log_10_(*p*-value). Candidate genes reaching suggestive or genome-wide significance are highlighted (see Table S8 for the complete list). (**D**) Quantile–quantile plot of observed vs. expected −log_10_(*p*-values) from the GWAS, to assess the inflation of the test statistics.

Parallel GWAS analyses were conducted on the two populations (141 ZL and 150 GIFT individuals) to map the resistance trait in the genome. Using the identified 4,421,935 SNPs, we analyzed population structure among the tilapia samples with the Admixture software (v 1.3) (Figure 9A). When the number of ancestral subgroups (*K*) = 2, most individuals were clearly separated into two populations, whereas higher *K* values revealed the multiple pedigree backgrounds of the ZL and GIFT lines. Principal component analysis (PCA) showed that PC1 (22.35%) and PC2 (6.28%) together explained a substantial proportion of the total genetic variance (Figure 9B). Most GIFT samples clustered tightly on the right side of the plot, while the ZL samples were more widely dispersed on the left. These patterns are consistent with the continuous strict selection for growth applied to GIFT and demonstrate that ZL has diverged genetically from GIFT after a prolonged period of selection for resistance.

Among the detected 4,421,935 SNPs, 4421 were significantly associated with the trait by GWAS (top 0.1% and *p* < 2.09 × 10^−6^), which localized 112 candidate genes after a manual check of gene functional information (Figure 9C,D). These genes were mainly involved in immune response, mucus nature, growth, and stress response (Table S8), indicating that the resistance of ZL is more of a trade-off between growth and immunity; it is also achieved by limiting excessive stress responses and mucus microbiota interactions [16,51,52]. Annotation of the localized genes suggested that they were employed in the different physiological processes in addition to their roles in immune regulation.

Comparison of the data outputs between multi-omics and GWAS revealed several candidate genes whose post-challenge expression changes were highly correlated with gill mucus microbiota abundance changes. It was found that nine and five significant SNPs (*p* < 2.78 × 10^−7^) were located in or near the *Flavobacterium*-correlated genes (as mentioned in Section 3.5; the same applies below) and *Hydrogenophaga*-correlated genes, respectively. Noticeably, three highly significant SNPs (*p* = 8.94 × 10^−6^, 7.24 × 10^−7^, and 7.74 × 10^−7^) identified the associated region from 36.746 to 36.748 Mb on LG10, just upstream of the *MMP13* (Collagenase 3) gene encoding. Expression of *MMP13* in the gill mucus of ZL was correlated with the abundance of *Flavobacterium* (*R* = 0.52, *p* = 0.004). It has been reported that *MMP13* is functional in mucin expression, production, or mucinous features through cooperation with mucin 1 and other mucins [53], which probably influenced the gill mucus environment and its resident microbiota. We also identified significant loci near the *Hydrogenophaga*-correlated genes. For example, a correlated locus (*R* = 0.77, *p* = 0.002) spanned the region of 14.278 to 14.280 Mb on LG16, including an upstream variant SNP (*p* = 9.60 × 10^−7^) and a synonymous variant SNP (*p* = 3.01 × 10^−5^) located in the *TUBA* (tubulin alpha chain) gene. *TUBA* encodes α-tubulin to participate in vesicle transportation and extracellular secretion [54] and contributed to the structure and motility of motile cilia on epithelial cells, which facilitate mucociliary clearance—the process of moving mucus and trapped particles or debris across the gill surface [55]. Another three significant SNPs (*p* = 3.47 × 10^−4^, 1.12 × 10^−5^, and 2.74 × 10^−7^) determined the candidate region from 31.243 to 31.247 Mb on LG7 where the *MAMDC4* (Thyroid hormone-induced protein B) gene is located. *MAMDC4* plays a crucial role in endosomal trafficking and impacts tight junctions and epithelial barrier function [56]. Moreover, *MAMDC4* has been reported to be regulated by bacterial colonization [57], an interesting interaction worth paying attention to. Taken together, the GWAS of the populations inferred a potential link between genes regulating the components of gill mucus and resistance to *S. agalactiae*.

## 4. Discussion

The present study demonstrates that the selectively bred tilapia ZL strain, combining *S. agalactiae* resistance with superior growth performance, is able to reshape its gill mucus microbiota and enrich *Flavobacterium*, *Hydrogenophaga*, and several other genera. The microbiota modulation appeared to be driven by heritable genetic variation in the host and was supported by differential gene expression, altered metabolic pathways, enhanced mucus synthesis and secretion, and improved mitochondrial respiration. These findings not only elucidate a unique resistance mechanism in ZL but also suggest that its distinctive gill mucus microbiota and associated compositional changes might help circumvent the typical growth–immunity trade-off observed in many teleosts’ selective breeding programs.

Although direct in vivo evidence of gill mucus microbiota antagonism against *S. agalactiae* is currently limited, our data outputs suggest a potential protective role for specific taxa, particularly *Flavobacterium*. Genomic analysis revealed that *Flavobacterium* possesses a polysaccharide utilization system capable of metabolizing high-molecular-weight polysaccharides [58]. Notably, previous studies have documented its ability to degrade the cell walls of *Streptococcus mutans*, a closely related pathogenic streptococcal species [59,60]. Moreover, in the resistant strain ZL, *Flavobacterium* rapidly dominated the gill microbiota and was accompanied by a sustained increase in acidophilic bacteria, particularly *Hydrogenophaga* and *Acidovorax* [61,62]. In contrast, these taxa only became prominent in the susceptible GIFT strain after the peak mortality phase. As *Flavobacterium* may produce organic acids during growth [63], the combined proliferation of these bacteria likely acidifies the gill mucus microenvironment. Since *S. agalactiae* has an optimal pH near 7.0 and is inhibited by certain organic acids [64,65], this shift may create conditions unfavorable for pathogen colonization and proliferation, thereby contributing to the resistance phenotype in ZL.

This microbial shift may be supported by potential metabolic interactions between *Flavobacterium* and *Hydrogenophaga*. Several organic acids (e.g., 3-indolepropionic acid, N6-(L-1,3-dicarboxypropyl)-L-lysine, and phenylacetylglycine) have been reported as potential carbon or nitrogen sources for *Hydrogenophaga* [61], while carbohydrates such as pseudaminic acid and α-1,5-L-arabinotriose could serve as substrates for *Flavobacterium* via its polysaccharide utilization loci [58,60]. *Flavobacterium* might contribute by secreting polysaccharide hydrolases that degrade flagellar components of Gram-negative bacteria (including *Hydrogenophaga*), thereby releasing utilizable organic acids and pseudaminic acid [66,67]. In turn, *Hydrogenophaga* could metabolize these compounds to support its growth. Such hypothetical cross-feeding may reduce direct competition between the two taxa, promoting their prolonged coexistence in the gill mucus and helping sustain the acidic microenvironment unfavorable to *S. agalactiae*.

Host transcriptomic profiling provided deeper mechanistic insights into the enhanced disease resistance observed in the ZL strain. Mfuzz temporal clustering of ZL-specific differentially expressed genes (DEGs) identified three dominant or accelerated expression clusters that were significantly enriched in pathways related to cytoskeleton organization in muscle cells, cardiac muscle contraction, motor proteins, adrenergic signaling in cardiomyocytes, glycolysis/gluconeogenesis, carbon metabolism, and calcium signaling. These enrichments suggest an accelerated epithelial secretory program and enhanced physiological preparedness in ZL compared with GIFT.

Genes whose expression positively correlated with *Flavobacterium* abundance in ZL were enriched in biological processes supporting mucus secretion and mucin glycosylation (e.g., SRP19, PPIB, FOXL1, LPAR4, ARPC3), immune and inflammatory regulation (e.g., FABP4A, PHF5A, SLC6B15, TRIM69, APOO, TRX1), cellular energy metabolism and mitochondrial function (e.g., NDUFA1/3/6/10/13, COX6B1/COX6C, TIMM50, PRDX3), and ribosome biogenesis and cell growth (e.g., LTV1, AK2) (Table S5). Similarly, genes correlated with *Hydrogenophaga* abundance fell into overlapping functional categories, including goblet cell differentiation, vesicular transport, mucin biosynthesis (e.g., HER6, TUBa2, SCFD1, CHST3a, NAGK), nutrient metabolism, antimicrobial substance secretion (e.g., GPD1c, NAGS, ARG1, UCK2b, AK2, ATP5MF, multiple MRPL genes), and immune modulation (e.g., IL10, PSME2, TXN, CH25h, DENR) (Table S7).

Integrated network analysis further revealed that these transcriptional responses are coordinated around mitochondrial function and energy metabolism. A prominent cluster included genes encoding mitochondrial ribosomal proteins (e.g., MRPL36, MRPS12, MRPL11), which were significantly upregulated during the *S. agalactiae* challenge [68]. This upregulation likely enhances mitochondrial protein synthesis, respiration, and biosynthetic activity in gill tissue, leading to elevated production of metabolites such as saccharopine (from lysine degradation), (R)-2,3-dihydroxy-3-methylbutanoate (from valine degradation), and 3-indolepropionic acid (a tryptophan metabolite). These metabolites can serve as carbon or nitrogen sources for *Flavobacterium* and *Hydrogenophaga*, thereby promoting their enrichment in the mucus.

Adjacent to this cluster, genes associated with the electron transport chain (e.g., NDUFA3/6/10/13, NDUFB6/7, NDUFS4 for Complex I; COX6B1, COX6C, COX7B2 for Complex IV) were also upregulated in ZL [69,70]. This coordinated increase in oxidative phosphorylation is expected to elevate succinate levels—an intermediate of the TCA cycle that acts as a signaling molecule in host–microbe interactions [71]. Additionally, upregulation of ATP6V1F, a subunit of vacuolar H^+^-ATPase, may support mitochondrial succinate synthesis while facilitating lysophospholipid production (e.g., LysoPE (20:4/0:0) and LysoPA (22:5/0:0)), which can modulate inflammatory responses and further shape the mucus microbiota [72,73]

Beyond metabolic support, a distinct group of genes influences the mucus environment directly. Upregulation of FOXL1, SRP19, and ARPC3 in ZL during challenge promotes goblet cell differentiation, mucin synthesis (v ia Wnt/β-catenin and SRP pathways), and vesicular transport/exocytosis for mucus secretion [74,75,76,77]. These changes likely alter the physicochemical properties of the gill mucus, creating a favorable niche that selectively enriches beneficial taxa such as *Flavobacterium* and *Hydrogenophaga* while enhancing barrier function and local immunity.

Taken together, these coordinated host responses—particularly in mucus properties, mitochondrial energy metabolism, and immune regulation—highlight strain-specific differences in host–microbiota interplay that appear largely absent in the GIFT strain. Such findings are consistent with the hypothesis that selective breeding for disease resistance in tilapia modulates both mucosal physiology and microbial community assembly, thereby contributing to improved health outcomes without the typical growth–immunity trade-off.

The importance of these host–microbiota interactions is further underscored by the broader context of fish microbiome research. Research on the fish microbiome has been extensively utilized to examine the impacts of diverse environmental factors, such as transportation stress [78], temperature changes [79], exposure to environmental toxins [80], and other abiotic stressors [81]. The mucosal surfaces of fish are in constant interaction with surrounding microbes and function as critical immune barriers while simultaneously serving as the primary habitat for diverse microbial communities [82]. A previous study in rainbow trout demonstrated that selective breeding programs may not only act on host genetic traits, but also favor distinct microbial assemblies that contribute to host resilience against pathogen invasion [83]. Extending these findings, our work further reveals that ZL can actively reshape its gill mucus microbiota through endogenous gene regulatory mechanisms. Our findings also align with the future research directions proposed by Morshed [84], who emphasized the need to investigate how commensal and pathogenic bacteria interact with mucosal immunity under environmental stress. Future studies should therefore examine the dynamic interplay between commensal and pathogenic bacteria with fish mucosal immunity under environmental effects, evaluate the efficacy of probiotic treatments in maintaining immune homeostasis in stressful conditions, assess how fish mucosal barriers respond to pathogens or their proteins during environmental stress, and elucidate the role of specific host genes or proteins in regulating microbiome homeostasis through targeted genetic manipulations.

While our findings contribute to this growing understanding, several methodological choices were made to ensure practical feasibility and reproducible strain-specific differences, yet they impose important limitations on data interpretation. The challenge model employed intraperitoneal injection at a high dose (5.8 × 10^8^ CFU/mL) to achieve uniform infection progression and synchronized sampling time points, which is difficult to obtain with natural gill or intestinal routes that typically produce highly variable disease kinetics among individuals. Although this approach reduces ecological realism compared with mucosal exposure, it was necessary to reliably discriminate responses between the two fish strains. Similarly, limited-volume gill samples were pooled across individuals to obtain sufficient biomass for both 16S rRNA sequencing and metabolomics analyses; while this strategy successfully revealed consistent population-level patterns, it precludes quantification of inter-individual variability and prevents direct one-to-one alignment of microbiota composition, metabolite profiles, and host transcriptome at the individual fish level. Consequently, Pearson correlation analyses between microbial taxa and gene expression were performed on mean values from pooled gill mucus samples, yielding a low effective sample size. These correlations therefore reflect macro-level exploratory associations rather than causal relationships. The same macro-level nature of the data, combined with sample-volume constraints, also made real-time qPCR validation of key differentially expressed genes or direct pathogen quantification in mucus impractical, especially as gene expression can vary markedly with culture conditions and current multi-omics approaches cannot establish definitive causal links at the individual gene level; high-depth RNA-seq data and internal consistency across omics datasets nonetheless provide sufficient support for the main findings. Overall, these trade-offs were deliberate to generate robust multi-omics insights under controlled conditions, but they highlight the need for future studies using individualized sampling and more ecologically relevant challenge models.

## 5. Conclusions

In this study, we applied a multi-omics approach to investigate a *Streptococcus agalactiae*-resistant strain of GIFT tilapia, referred to as ZL. Our results revealed that ZL distinctly remodels its gill mucus microbiota in response to infection. This microbiota restructuring appears to confer a survival advantage by limiting gill-to-gill transmission of the pathogen, a hypothesis supported by our GWAS findings. These observations provide a striking example of how host–microbiota interactions contribute to immune defense mechanisms in fish. Overall, our findings establish a robust foundation for future studies exploring microbiota-based strategies to enhance disease resistance in aquaculture species.

## Data Availability

The 16S rRNA gene sequencing data and gill transcriptome data generated in this study have been deposited in the Genome Sequence Archive (GSA) of the National Genomics Data Center (https://ngdc.cncb.ac.cn/gsa (accessed on 10th April 2026)) under BioProject accession PRJCA025004.

## Supplementary Materials

The following supporting information can be downloaded at https://www.mdpi.com/article/10.3390/ani16091389/s1: Figure S1: Pipelines of the DEG identification.; Figure S2: Overview of ZL-specific DEGs during the *S. agalactiae* challenge; Figure S3: Minimum centroid distance (dmin) as a function of cluster number (k) for Mfuzz soft clustering analysis. Figure S4: Microbial alpha- and beta-diversity in intestine contents during the *S. agalactiae* challenge. Figure S5: Composition and relative abundance of microbiota in intestine contents; Figure S6: Discriminated microbiota identified by the LEfSe Analysis. Table S1: Abundance of microbiota at class level; Table S2: Abundance of microbiota at genus level. Table S3: Detailed Gene Members of the 12 Clusters from 582 DEGs; Table S4: Correlationship between gene expression and abundence of Flavobacterium during the challenge; Table S5: Annotation of the involved genes in function network of Flavobacterium; Table S6: Correlationship between gene expression and abundence of Hydrogenophaga during the challenge; Table S7: Annotation of the involved genes in function network of Hydrogenophaga; Table S8: Candidate genes localized by GWAS. The following references are cited in the Supplementary Materials: [24,33,45,46,47,48,49,85,86,87,88,89,90].

## Abbreviations

The following abbreviations are used in this manuscript:

ZL	Zhuangluo 1
GIFT	Genetically Improved Farmed Tilapia
GWAS	genome-wide association study
