# Supplementary material for: Identification of Association Between Mucus Microbiota and Gene Expression in the Gill of a Streptococcus agalactiae-Resistant Nile Tilapia Strain Though Multi-Omics Analyses
Source: Animals (Basel). 2026 May 2;16(9):1389. doi: 10.3390/ani16091389 (PMC13163006; doi:10.3390/ani16091389)
Supplement: Supplementary file 1 [file animals-16-01389-s001.zip › SupplementaryNotesAndFigures.pdf]

Supplementary notes and figures

Identification of association between mucus microbiota and gene expression in the gill of a Streptococcus-agalactiae-resistant Nile tilapia train though multi-omics analyses

1. Supplementary notes

1.1 SNP Calling and Genome-Wide Association Study (GWAS) Methods

All tilapia specimens used in this study were sourced from the Freshwater Fish Breeding Center of the Guangxi Academy of Fishery Sciences. Following an outbreak of Streptococcus agalactiae in the summer of 2023, we selected 141 individuals of the "Zhuangluo 1" tilapia strain, which exhibited stable traits and resistance to *S. agalactiae*, along with 150 GIFT tilapia as a control group. For sampling, blood was collected from the resistant "Zhuangluo 1" individuals, while tail fin clips were obtained from moribund fish. Samples were immediately flash-frozen in liquid nitrogen and stored at -80°C until processing. Genomic DNA was extracted from muscle tissue using the Qiagen DNeasy Blood and Tissue Kit (Qiagen, CA, USA). Sequencing libraries were constructed according to Illumina's high-throughput sequencing protocol, and whole-genome resequencing was performed to generate paired-end reads (PE-150) with an average sequencing depth of 5.9×.

Raw sequencing reads were subjected to adapter trimming and quality control prior to alignment to the reference genome. Multi-sample joint variant calling was then performed to identify sequence variations, including single nucleotide polymorphisms (SNPs). Specifically, Cutadapt software[1] was used for preprocessing the raw reads, which involved removing adapter sequences and filtering out low-quality reads (e.g., those with Phred scores below a predefined threshold, typically Q20 or higher, to ensure reliable base calls).

Following preprocessing, the cleaned reads were aligned to the GIFT tilapia reference genome [2] using BWA (Burrows-Wheeler Aligner) software [3]. This alignment step employed the BWA-MEM algorithm, which is optimized for handling longer reads and accounting for potential insertions, deletions, and structural variations. Post-alignment, duplicate reads arising from PCR amplification were identified and marked using the MarkDuplicates module in the GATK (Genome Analysis Toolkit) software package [4]. This step is crucial to avoid inflating variant calls due to technical artifacts.

Variant calling was conducted using GATK's HaplotypeCaller in GVCF mode for each sample individually, followed by joint genotyping across all samples with GenotypeGVCFs. This approach leverages local de novo assembly around potential variant sites to improve accuracy, particularly in regions with indels or complex haplotypes. The resulting variant calls were consolidated into a single Variant Call Format (VCF) file, representing the initial set of variants for downstream analysis.

1.2 Optimization of Variant Calls Using Machine Learning

To enhance the accuracy of the variant dataset, we applied GATK's Variant Quality Score Recalibration (VQSR) method, which utilizes machine learning to recalibrate variant quality scores based on a training set of known variants. This process systematically filters out false positives while retaining true variants. The workflow began with the generation of an initial VCF file from the duplicate-marked BAM files using samtools mpileup and call commands [5]. This preliminary VCF, containing putative SNPs, served as the "known sites" dataset for model training. These known SNPs were input into GATK's VariantRecalibrator tool, which builds a Gaussian mixture model based on annotations such as mapping quality (MQ), quality by depth (QD), Fisher strand bias (FS), and strand odds ratio (SOR). The model assigns a recalibrated quality score (VQSLOD) to each variant in the original dataset, enabling probabilistic filtering.

Variants were then filtered using ApplyVQSR to retain those passing a sensitivity tranche (99.0%), ensuring high recall of true positives. Post-recalibration, additional quality control was performed using VCFtools [6] with parameters "--max-missing 0.8 --maf 0.05". This excluded individuals with genotype missingness exceeding 20% and variants with minor allele frequency (MAF) below 0.05, as low-MAF sites are prone to sequencing errors and may not be informative for association studies. The filtered VCF file was subjected to genotype imputation using Beagle version 4.1 [7] to fill in missing genotypes based on linkage disequilibrium patterns within the population. This resulted in a high-quality SNP and short indel genotype matrix suitable for GWAS. Quality control and filtering were then applied using PLINK version 1.90 [8]. Specifically, variants with MAF < 0.05 were removed, as were those deviating from Hardy-Weinberg equilibrium (HWE) at a threshold of $P < 0.001$. Individuals with excessive missingness (e.g., >5%) were also excluded to maintain dataset integrity.

1.3 Genome-Wide Association Analysis

GWAS was conducted using a classical approach with the EMMAX software [9] and its Efficient Mixed-Model Association eXpedited (EMMAX) method to compare the study populations and identify chromosomal regions associated with the target trait (*S. agalactiae* resistance). The LMM accounts for population structure and cryptic relatedness, reducing false positives due to confounding factors. The analysis computed association statistics for each variant, yielding P-values adjusted for the mixed model. To visualize results, Manhattan plots (with -log10(P) on the y-axis) and quantile-quantile (QQ) plots were generated using the qqman package in R [10].

1.4 16s rRNA analysis for intestinal content of ZL & GIFT

Alpha diversity of intestine microbiota was obviously lower than that in gill mucus. The calculated Shannon, Shannon even, and Simpson indexes indicated two similar increases in both GIFT and ZL from 0 to 12 h and from 0 to 48 h, respectively (Fig. S3A). Beta diversity analysis also indicates the robustness of intestinal microbiota in most sample (Fig. S3B&C). The total abundance of the main classes, Bacteroidia and Fusobacteriia, was always stable at over 90% in ZL but considerably fluctuated in GIFT. The rise and decline of Bacilli in GIFT were respectively accompanied by the decline of Bacteroidia at 12 h and rise of Actinobacteria and Alphaproteobacteria at 48 h (Fig. S4A). The identified genera in intestine contents exhibited that abundance variation of each class depended on a couple of dominant genera, like *Cetobacterium* of Fusobacterii, *Plesiomonas* of Gammaproteobacteri, *Romboutsia* of Clostridiaz, and *Streptococcus* of Bacilli (Fig. S4B). It was observed that the abundance of *Streptococcus* in GIFT is nearly 9 time

that in ZL at the acute phase of infection (12 h post the infection), while *Romboutsi* increased by 6.73% in ZL, comparing to only increased by 0.25% in GIFT. The relative abundance of *Romboutsia* has been found to be associated with reducing severity of pancreatitis and associated sepsis [11,12]. Referencing the demonstrated view that Clostridia are able to inhibit growth of pathogens by competing for the adhesion sites in hosts' upper intestine [13], there might be a potential relationship between recovery from the infection and existence of Clostridia. But overall, the microbiota diversity in intestine contents is much lower than that in gill mucus, also determined by the beta diversity analysis, which suggested the intestine only had a minor disturbance during the challenge.

LEfSe analysis demonstrated overall robustness in the intestinal microbiota composition across the studied groups (Fig. S5). Although differentially abundant genera were identified among the four groups, only Streptococcus in the Gi12h group exerted a substantial influence on the overall intestinal microbiota structure. While certain bacteria showed differential abundance in the ZLi0h and ZLi12h groups, these could not be reliably assigned at the genus level. Moreover, their low relative abundance likely limits their potential biological impact. Different from gill mucus, LefSE failed to identify many discriminate genera but only three. They are *Streptococcus* in Gi12h, *Romboutsia* (6.73%) and *Turicibacter* (0.68%) in ZLi12h. But considering the relative abundance of *Turicibacter* and *Turicibacter* was below 1%, its influence in intestine microbiota is limited. If further study provides more evidence, *Romboutsia* could be a candidate bacteria genus for similar research.

References

1. Martin, M. Cutadapt Removes Adapter Sequences from High-Throughput Sequencing Reads. *EMBnet. journal* **2011**, *17*, 10–12.
2. Etherington, G.J.; Nash, W.; Ciezarek, A.; Mehta, T.K.; Barria, A.; Peñaloza, C.; Khan, M.G.Q.; Durrant, A.; Forrester, N.; Fraser, F.; et al. Chromosome-Level Genome Sequence of the Genetically Improved Farmed Tilapia (GIFT, Oreochromis Niloticus) Highlights Regions of Introgression with O. Mossambicus. *BMC Genomics* **2022**, *23*, doi:10.1186/s12864-022-09065-8.
3. Li, H.; Durbin, R. Fast and Accurate Short Read Alignment with Burrows–Wheeler Transform. *Bioinformatics* **2009**, *25*, 1754, doi:10.1093/BIOINFORMATICS/BTP324.
4. McKenna, A.; Hanna, M.; Banks, E.; Sivachenko, A.; Cibulskis, K.; Kernytsky, A.; Garimella, K.; Altshuler, D.; Gabriel, S.; Daly, M.; et al. The Genome Analysis Toolkit: A MapReduce Framework for Analyzing next-Generation DNA Sequencing Data. *Genome Res.* **2010**, *20*, 1297, doi:10.1101/GR.107524.110.
5. H, L.; B, H.; A, W.; T, F.; J, R.; N, H.; G, M.; G, A.; R, D. The Sequence Alignment/Map Format and SAMtools. *Bioinformatics* **2009**, *25*, 2078–2079, doi:10.1093/BIOINFORMATICS/BTP352.
6. Danecek, P.; Auton, A.; Abecasis, G.; Albers, C.A.; Banks, E.; DePristo, M.A.; Handsaker, R.E.; Lunter, G.; Marth, G.T.; Sherry, S.T.; et al. The Variant Call Format and VCFtools. *Bioinformatics* **2011**, *27*, 2156, doi:10.1093/BIOINFORMATICS/BTR330.
7. Browning, B.L.; Tian, X.; Zhou, Y.; Browning, S.R. Fast Two-Stage Phasing of Large-Scale Sequence Data. *Am. J. Hum. Genet.* **2021**, *108*, 1880–1890, doi:10.1016/j.ajhg.2021.08.005.
8. Purcell, S.; Neale, B.; Todd-Brown, K.; Thomas, L.; Ferreira, M.A.R.; Bender, D.; Maller, J.; Sklar, P.; De Bakker, P.I.W.; Daly, M.J.; et al. PLINK: A Tool Set for Whole-Genome Association and Population-Based Linkage Analyses. *Am. J. Hum. Genet.* **2007**, *81*, 559–575, doi:10.1086/519795.

9. Kang, H.M.; Sul, J.H.; Service, S.K.; Zaitlen, N.A.; Kong, S.Y.; Freimer, N.B.; Sabatti, C.; Eskin, E. Variance Component Model to Account for Sample Structure in Genome-Wide Association Studies. *Nat. Genet.* **2010**, *42*, 348–354, doi:10.1038/ng.548.
10. Turner, S.D. Qqman: An R Package for Visualizing GWAS Results Using QQ and Manhattan Plots. *Biorxiv* **2014**, 005165.
11. Gerritsen, J.; Fuentes, S.; Grievink, W.; van Niftrik, L.; Tindall, B.J.; Timmerman, H.M.; Rijkers, G.T.; Smidt, H. Characterization of Romboutsia Ilealis Gen. Nov., Sp. Nov., Isolated from the Gastro-Intestinal Tract of a Rat, and Proposal for the Reclassification of Five Closely Related Members of the Genus Clostridium into the Genera Romboutsia Gen. Nov., Intestinib. *Int. J. Syst. Evol. Microbiol.* **2014**, *64*, 1600–1616, doi:10.1099/ijs.0.059543-0.
12. Gerritsen, J. The Genus Romboutsia: Genomic and Functional Characterization of Novel Bacteria Dedicated to Life in the Intestinal Tract, Wageningen University and Research, 2015.
13. Mohapatra, S.; Chakraborty, T.; Kumar, V.; Deboeck, G.; Mohanta, K.N. Aquaculture and Stress Management: A Review of Probiotic Intervention. *J. Anim. Physiol. Anim. Nutr. (Berl).* 2013, *97*, 405–430.

2. Supplementary figures

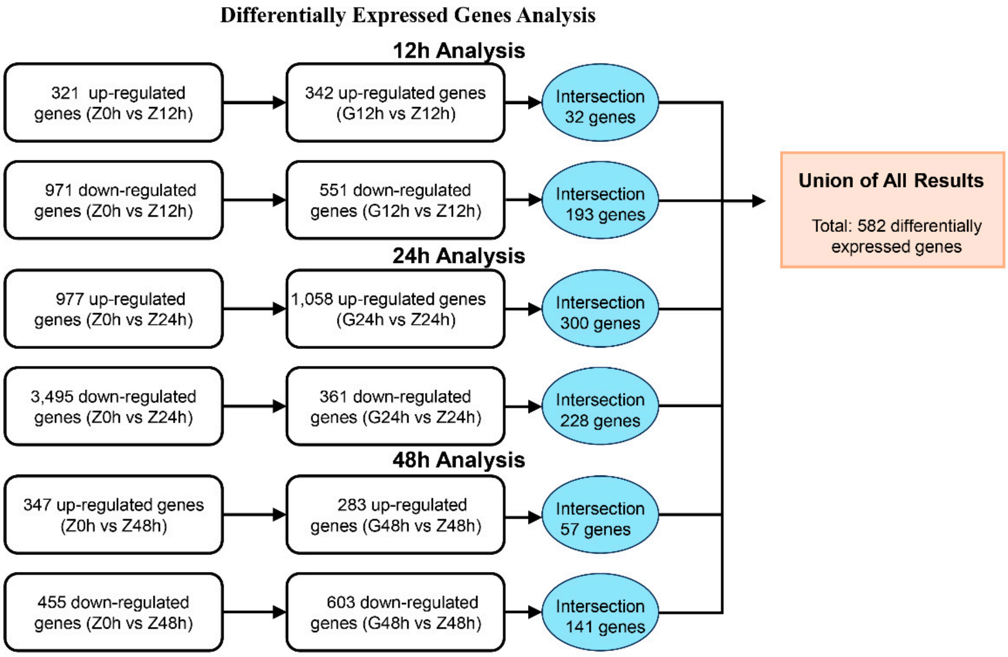

Supplementary Fig. 1 **Pipelines of the DEG identification.**

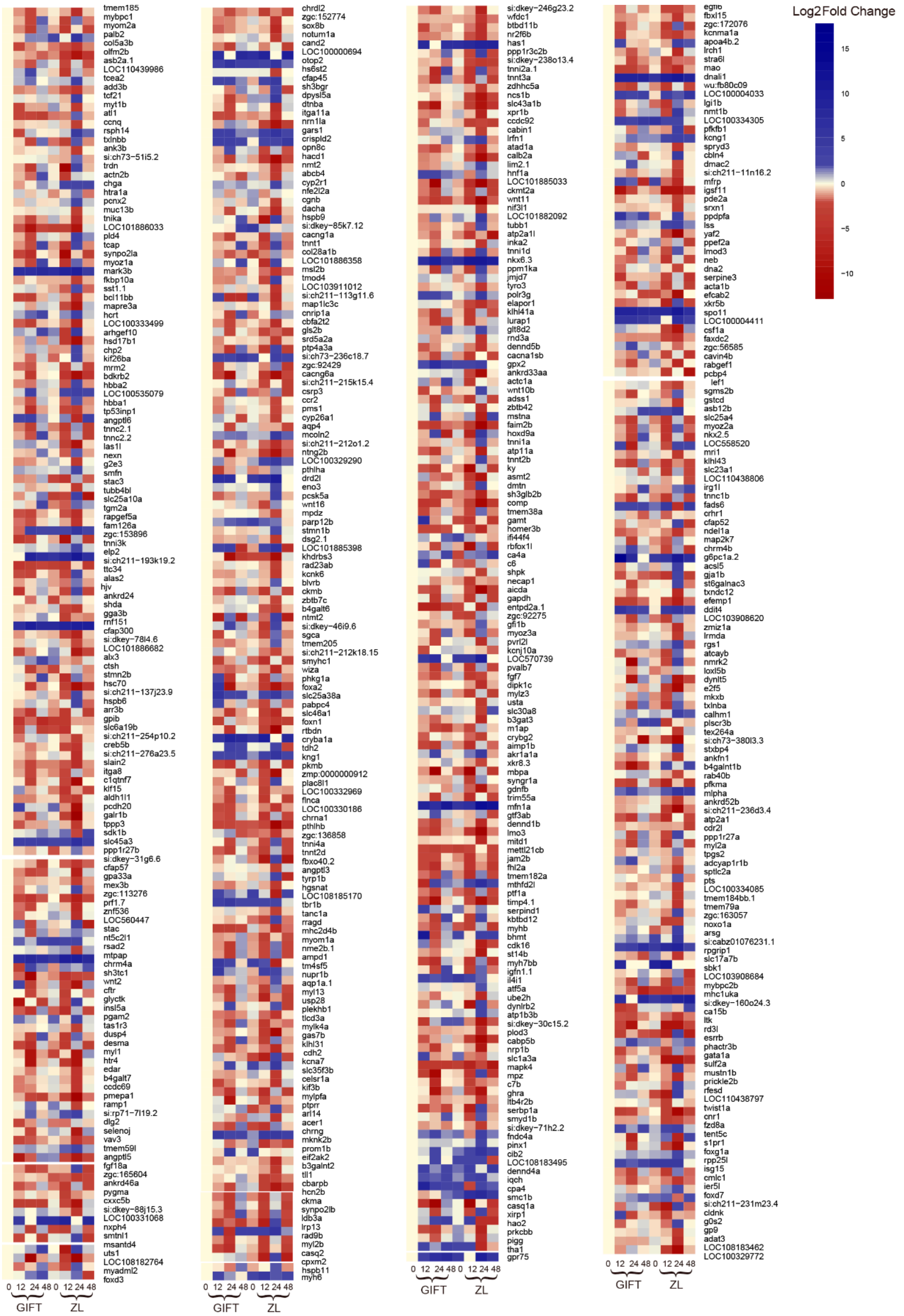

Supplementary Fig. 2 **Overview of ZL-specific DEGs during the *S. agalactiae* challenge.** Each row corresponds to a gene and each column for a time point in gill of GIFT/ZL, respectively. The colors indicate the Log2 fold change of FPKM compared to GIFT in 0h. The symbols of the genes was listed on the right.

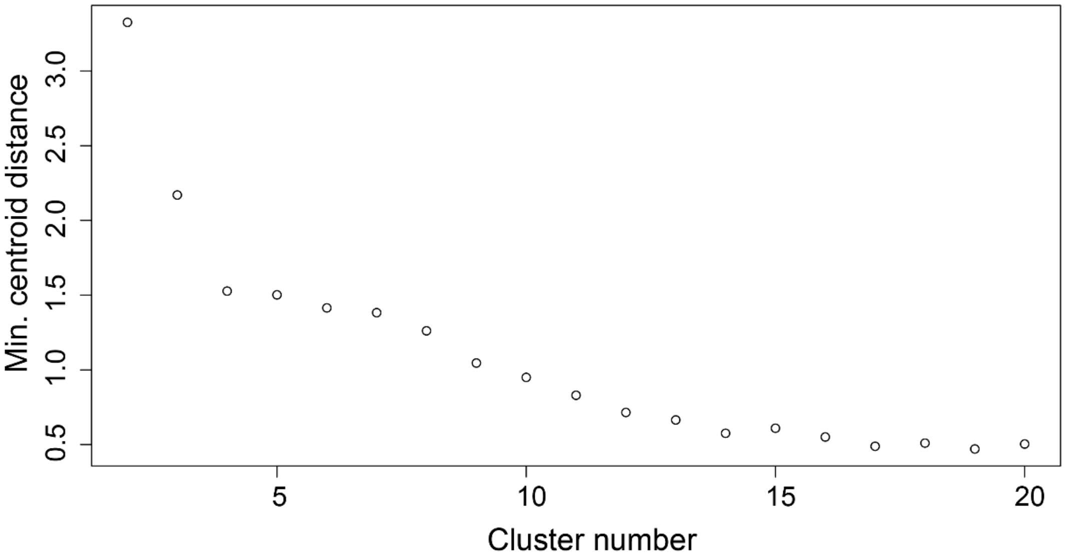

Supplementary Fig. 3 Minimum centroid distance (dmin) as a function of cluster number (k) for Mfuzz soft clustering analysis. The plot shows the dmin as a function of cluster number (k) ranging from 2 to 20. The elbow point at k = 12 was selected as the optimal number of clusters, where the rate of decrease in dmin begins to plateau, indicating diminishing returns in cluster separation with additional clusters.

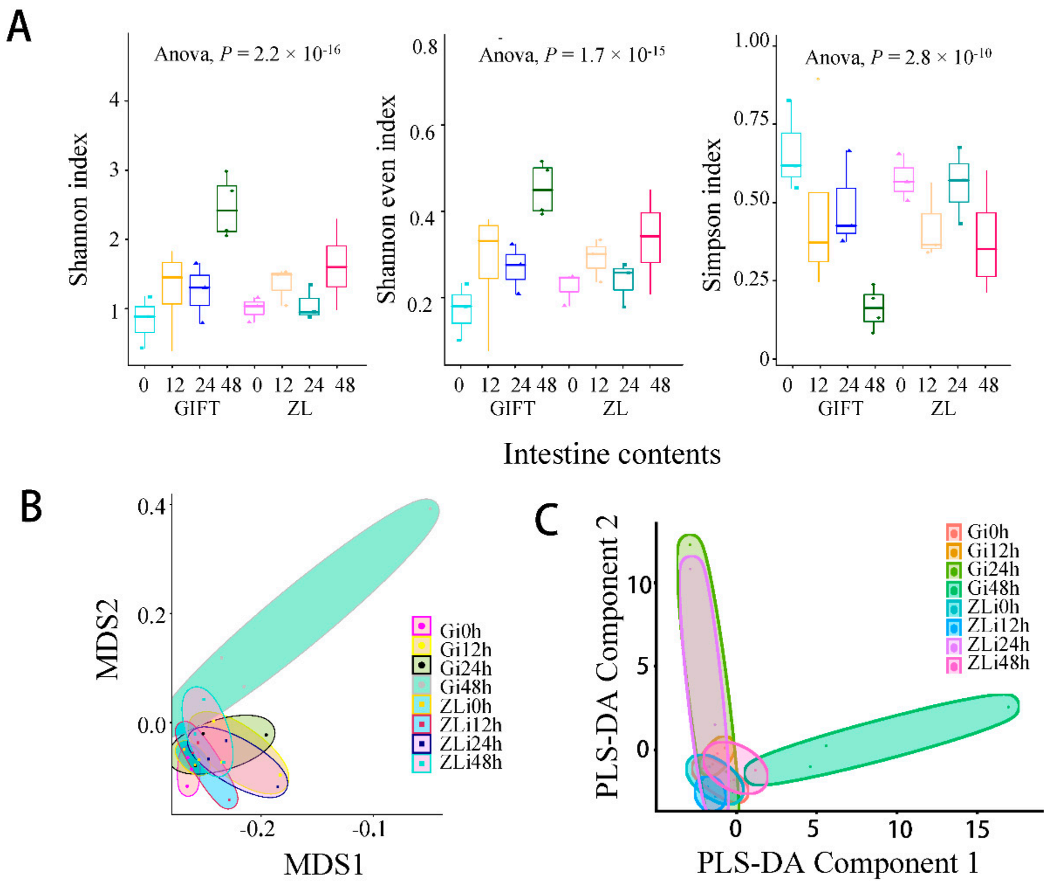

Supplementary Fig. 4 **Microbial alpha- and beta-diversity in intestine contents during the *S. agalactiae* challenge.** (A) Box plots of microbial alpha-diversity indices (Shannon index, Shannon evenness index, Simpson index) in intestine contents of the two tilapia strains at 0, 12, 24, and 48 hours post the challenge. *P* values from ANOVA reflect significant differences in alpha-diversity among groups. (B) Non-metric multidimensional scaling (NMDS) ordination illustrating beta-diversity and microbial community structure in intestine contents (right) across time points. (C) Partial least squares discriminant analysis (PLS-DA) scores plot showing differentiation in microbial community structure between tilapia lines and across time points in gill mucus (left) and intestine contents (right). Abbreviations of the sample IDs in (B) and (C): G for GIFT; i for intestine contents.

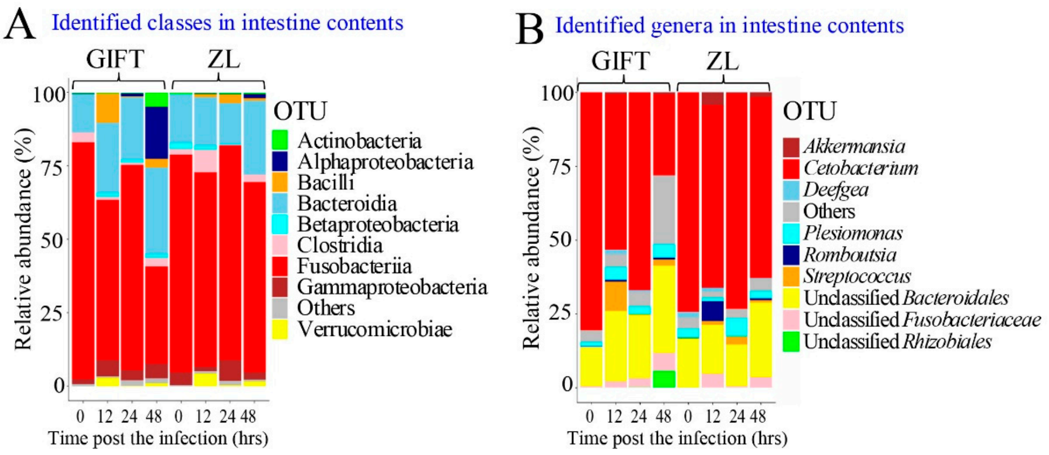

Supplementary Fig. 5 **Composition and relative abundance of microbiota in intestine contents.** (A) and (B) Identified classes and genera in intestine contents, respectively. Microbiota with less than 5 % abundance were categorized as “others”.

A

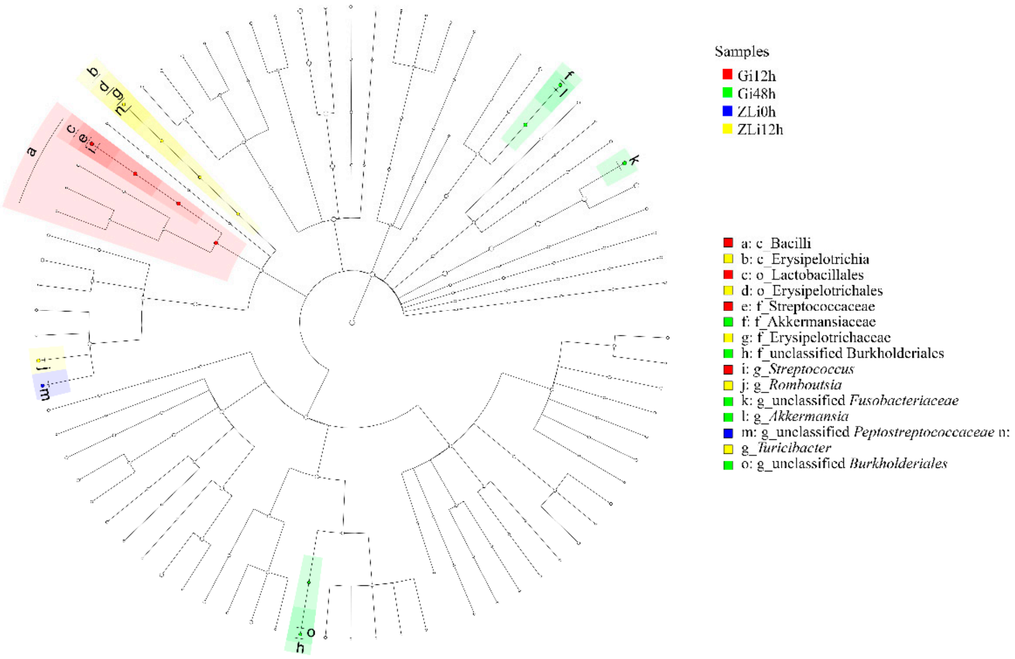

Supplementary Fig. 6 **Discriminated microbiota identified by the LEfSe Analysis.** Circos visualization of bacteria composition in intestine contents (The concentric circles (inner to outer) represent ranks (phylum to genus). Colors correspond to group-specific enriched taxa; links between circles connect taxa to their relative abundance across groups (values on circles = abundance), and the right legend lists significantly differential taxa (e.g., C. butyricum, L. fermentum). Abbreviations of the sample IDs: G for GIFT; i for intestine contents.
