# Supplementary material for: Identification of Association Between Mucus Microbiota and Gene Expression in the Gill of a Streptococcus agalactiae-Resistant Nile Tilapia Strain Though Multi-Omics Analyses
Source: Animals (Basel). 2026 May 2;16(9):1389. doi: 10.3390/ani16091389 (PMC13163006; doi:10.3390/ani16091389)
Supplement: Supplementary file 1 [file animals-16-01389-s001.zip › Fig.S1 DEG selection.pdf]

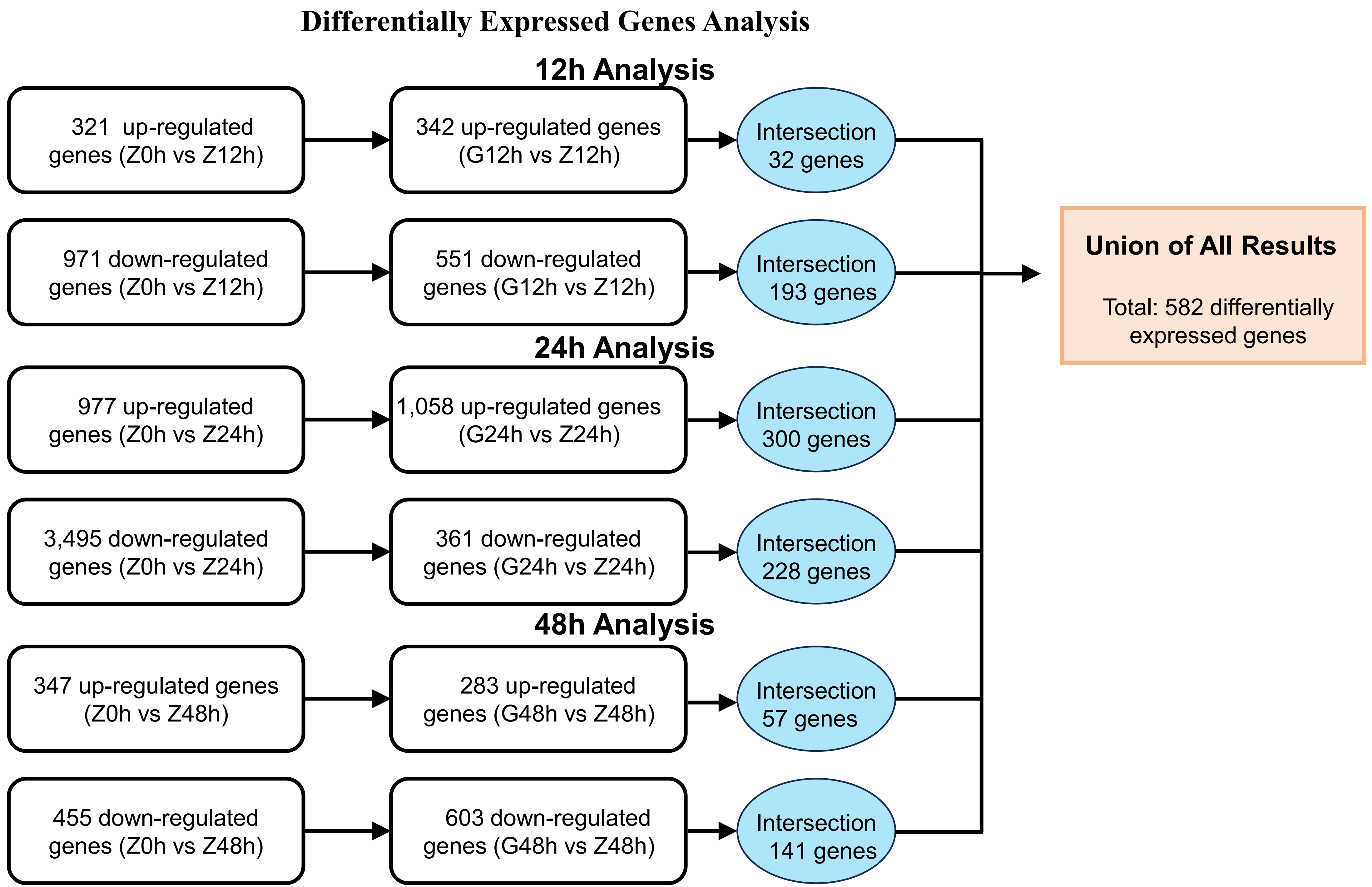
Differentially Expressed Genes Analysis
12h Analysis
321 up-regulated genes (Z0h vs Z12h)
342 up-regulated genes (G12h vs Z12h)
Intersection 32 genes
971 down-regulated genes (Z0h vs Z12h)
551 down-regulated genes (G12h vs Z12h)
Intersection 193 genes
24h Analysis
977 up-regulated genes (Z0h vs Z24h)
1,058 up-regulated genes (G24h vs Z24h)
Intersection 300 genes
3,495 down-regulated genes (Z0h vs Z24h)
361 down-regulated genes (G24h vs Z24h)
Intersection 228 genes
48h Analysis
347 up-regulated genes (Z0h vs Z48h)
283 up-regulated genes (G48h vs Z48h)
Intersection 57 genes
455 down-regulated genes (Z0h vs Z48h)
603 down-regulated genes (G48h vs Z48h)
Intersection 141 genes
Union of All Results
Total: 582 differentially expressed genes
