# Supplementary material for: Identification of Association Between Mucus Microbiota and Gene Expression in the Gill of a Streptococcus agalactiae-Resistant Nile Tilapia Strain Though Multi-Omics Analyses
Source: Animals (Basel). 2026 May 2;16(9):1389. doi: 10.3390/ani16091389 (PMC13163006; doi:10.3390/ani16091389)
Supplement: Supplementary file 1 [file animals-16-01389-s001.zip › Fig.S6 intestinal LefSe.pdf]

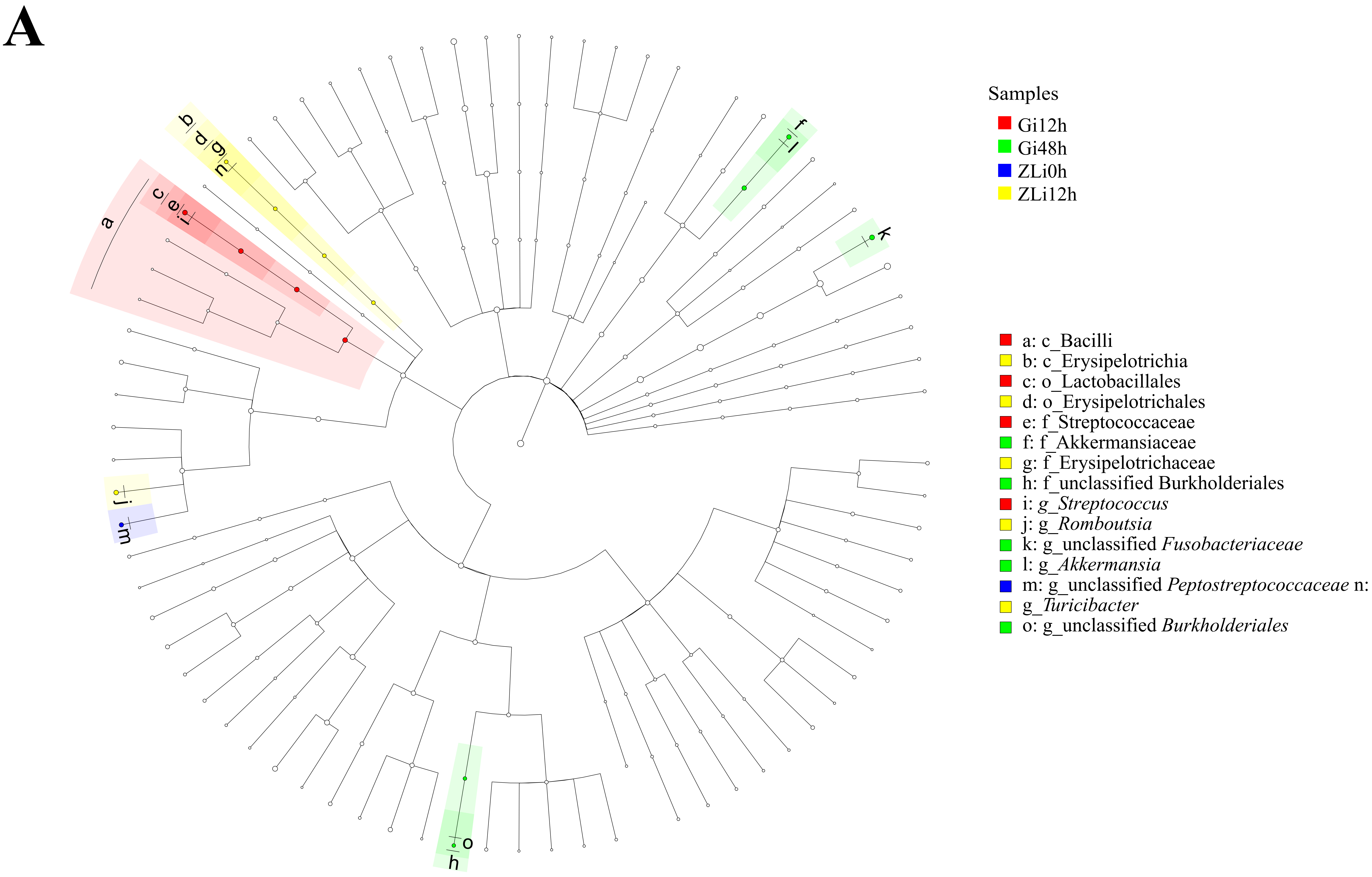
A
Samples
Gi12h
Gi48h
ZLi0h
ZLi12h
a: c_Bacilli
b: c_Erysipelotrichia
c: o_Lactobacillales
d: o_Erysipelotrichales
e: f_Streptococcaceae
f: f_Akkermansiaceae
g: f_Erysipelotrichaceae
h: f_unclassified Burkholderiales
i: g_*Streptococcus*
j: g_*Romboutsia*
k: g_unclassified *Fusobacteriaceae*
l: g_*Akkermansia*
m: g_unclassified *Peptostreptococcaceae* n:
g_*Turicibacter*
o: g_unclassified *Burkholderiales*
