# Supplementary figures and images for: Identification of Association Between Mucus Microbiota and Gene Expression in the Gill of a Streptococcus agalactiae-Resistant Nile Tilapia Strain Though Multi-Omics Analyses
Source: Animals (Basel). 2026 May 2;16(9):1389. doi: 10.3390/ani16091389 (PMC13163006; doi:10.3390/ani16091389)

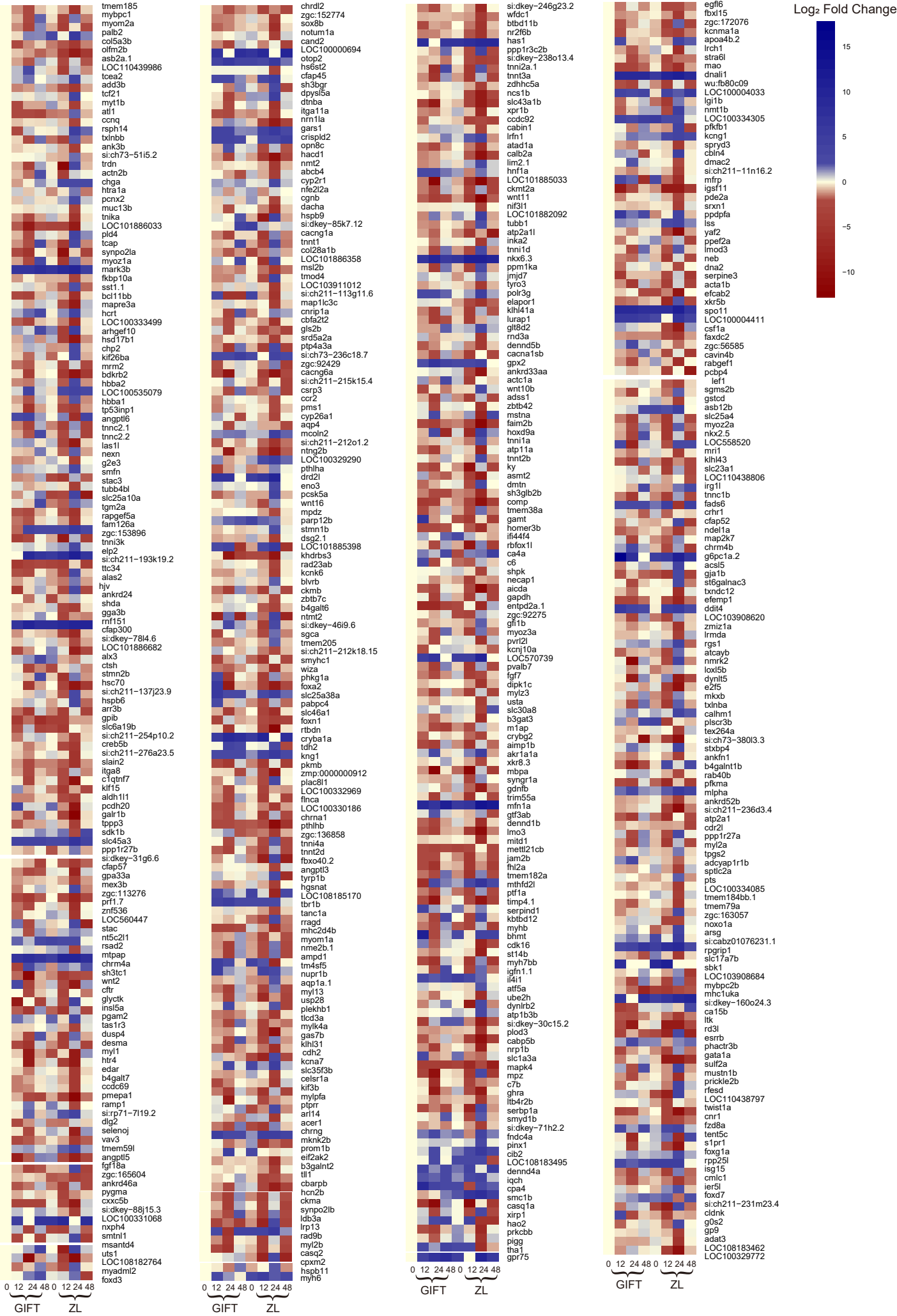

Supplement: Supplementary file 1 [file animals-16-01389-s001.zip › Fig.S2 Gene_Heatmap.pdf]

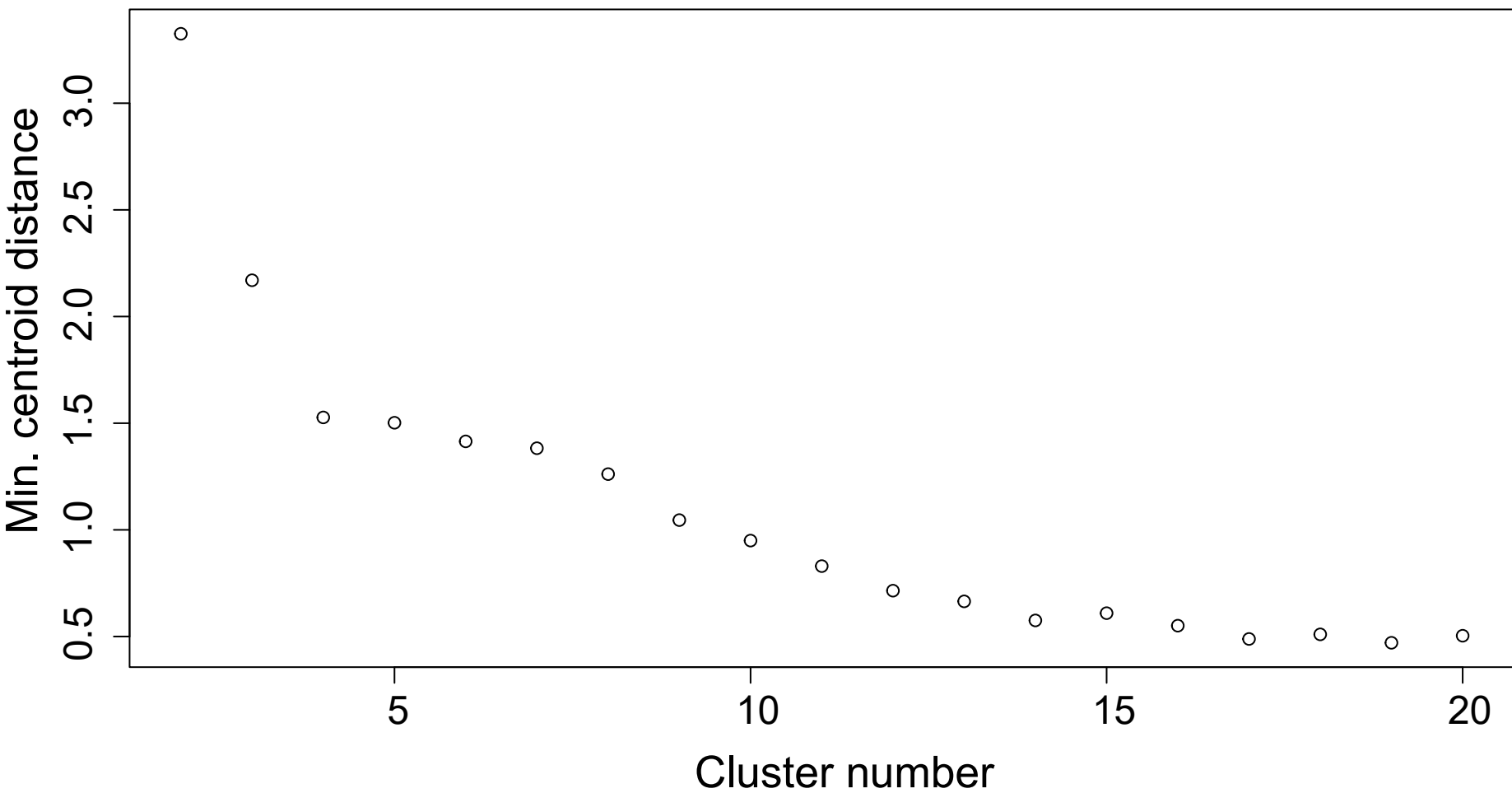

Supplement: Supplementary file 1 [file animals-16-01389-s001.zip › Fig.S3 dmin.pdf]

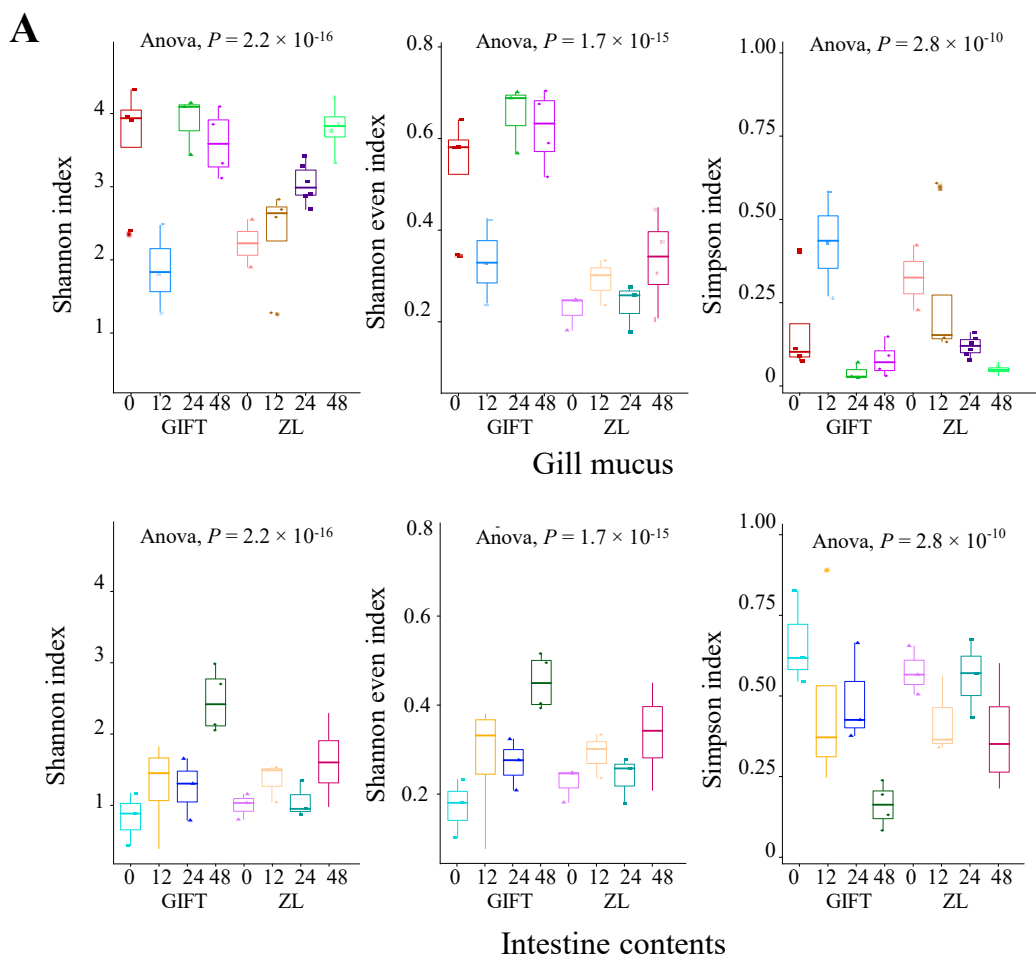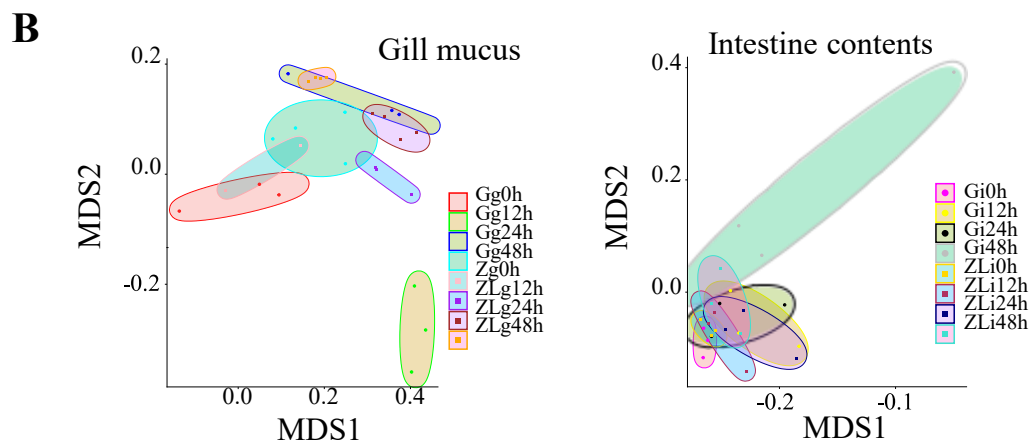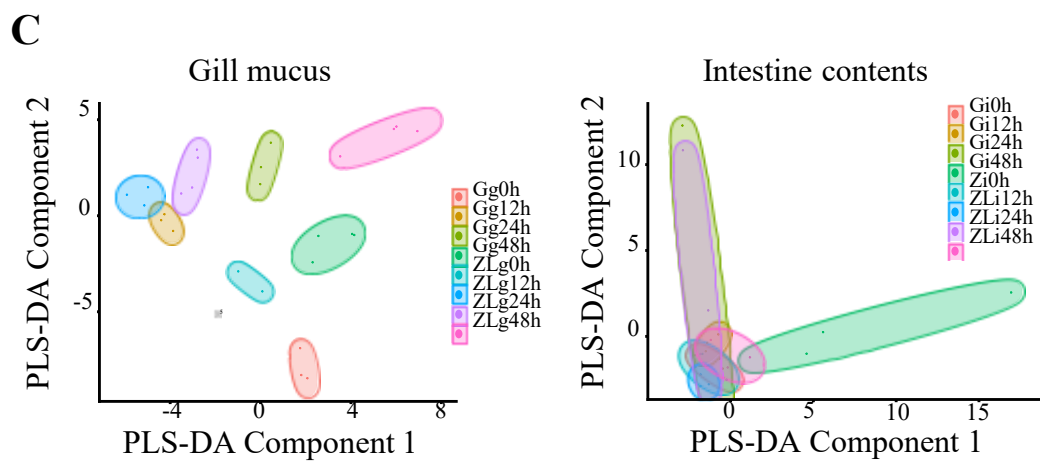

Supplement: Supplementary file 1 [file animals-16-01389-s001.zip › Fig.S4 intestinal ALPHA_BETA.pdf]

## A Identified classes in intestine contents

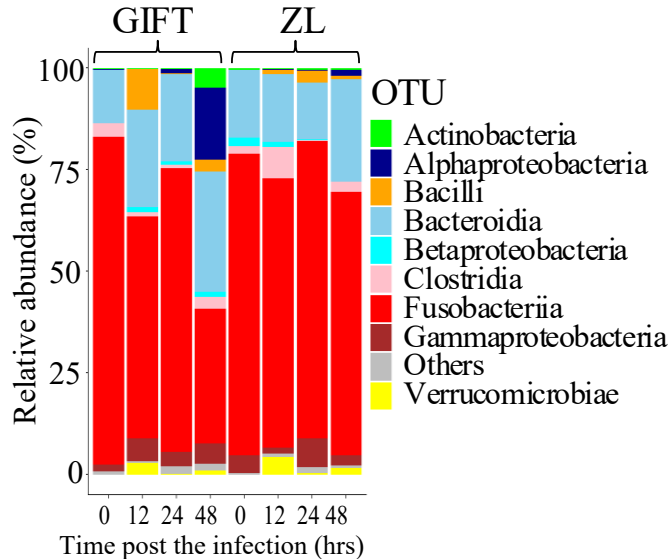

## B Identified genera in intestine contents

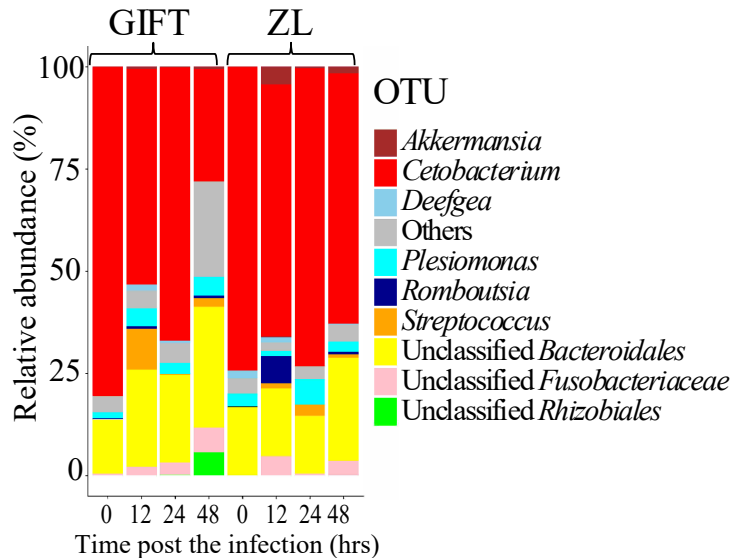

Supplement: Supplementary file 1 [file animals-16-01389-s001.zip › Fig.S5 intestinal abundance.pdf]
